# ATR inhibition enables complete tumour regression in ALK-driven NB mouse models

**DOI:** 10.1038/s41467-021-27057-2

**Published:** 2021-11-24

**Authors:** Joanna Szydzik, Dan E. Lind, Badrul Arefin, Yeshwant Kurhe, Ganesh Umapathy, Joachim Tetteh Siaw, Arne Claeys, Jonatan L. Gabre, Jimmy Van den Eynden, Bengt Hallberg, Ruth H. Palmer

**Affiliations:** 1 Department of Medical Biochemistry and Cell Biology, Institute of Biomedicine, Sahlgrenska Academy, University of Gothenburg, SE-40530 Gothenburg, Sweden; 2 Department of Human Structure and Repair, Anatomy and Embryology Unit, Ghent University, 9000 Ghent, Belgium

**Keywords:** Growth factor signalling, Paediatric cancer, Checkpoint signalling

## Abstract

High-risk neuroblastoma (NB) often involves *MYCN* amplification as well as mutations in *ALK*. Currently, high-risk NB presents significant clinical challenges, and additional therapeutic options are needed. Oncogenes like *MYCN* and *ALK* result in increased replication stress in cancer cells, offering therapeutically exploitable options. We have pursued phosphoproteomic analyses highlighting ATR activity in ALK-driven NB cells, identifying the BAY1895344 ATR inhibitor as a potent inhibitor of NB cell growth and proliferation. Using RNA-Seq, proteomics and phosphoproteomics we characterize NB cell and tumour responses to ATR inhibition, identifying key components of the DNA damage response as ATR targets in NB cells. ATR inhibition also produces robust responses in mouse models. Remarkably, a 2-week combined ATR/ALK inhibition protocol leads to complete tumor regression in two independent genetically modified mouse NB models. These results suggest that NB patients, particularly in high-risk groups with oncogene-induced replication stress, may benefit from ATR inhibition as therapeutic intervention.

## Abstract (web summary)

Effective therapeutic options are still needed in neuroblastoma treatment. Here, the authors, through a comprehensive proteomics analysis, identify ATR as a potential therapeutic target of neuroblastoma and demonstrate the efficacy of the ATR inhibitor BAY1895344 in combination with the ALK tyrosine kinase inhibitor lorlatinib.

## Introduction

Neuroblastoma (NB) is a childhood cancer arising from the neural crest that accounts for 15% of all paediatric tumour-related deaths^1^. Aggressive NB often responds to initial treatment, but later relapses represent a major clinical problem with poor prognosis and survival rates of around 35%^2,3^. Understanding the underlying molecular mechanisms leading to initiation and progression of NB is important to identify actionable therapeutic targets and pathways involved in this pathogenesis.

NB characteristically has few mutations, but instead exhibits a high number of somatic chromosomal lesions at the genetic level, including structural variations (SVs) and copy number alterations (CNAs)^4–6^. Genes with increased somatic mutation frequencies in NB include *ALK*, *PTPN11*, *ATRX* and *NRAS*^4,6^, while common genetic features include deletion of chromosome arm 1p, gain of parts of 17q, and aneuploidy^7,8^. Amplification of the *MYCN* transcription factor is observed in approximately 20% of NB and is an important predictive marker of patient survival^9,10^. Gain of chromosome 2p, so called “2p gain”, often results in increased copy numbers of *ALK*, *MYCN* and the *ALKAL2* ALK ligand^11^. Deletion of chromosome 11q, which disrupts *DLG2* and *SHANK2* as well as the DNA damage response (DDR) genes *ATM*, *CHK1*, *MRE11* and *H2AFX*, represents an additional important chromosomal aberration in NB that is used to distinguish between high- and low-risk cases^4,6,12–18^. In addition, chromothripsis has also been described in NB^19^.

Such chromosomal lesions in NB result in increased genome instability, necessitating maintenance of genome integrity by DNA damage sensor mechanisms that arrest the cell cycle by the activation of downstream signalling pathways. Two important sensors involve the PI3-kinase-related protein kinases (PIKKs), ataxia telangiectasia mutated (ATM) and ataxia telangiectasia and Rad3-related (ATR), which regulate CHK2 and CHK1, respectively^20–22^. ATM senses double-strand breaks, whereas ATR is activated by single-stranded DNA^23^. ATR has an important role in cell survival in response to replication stress, by preventing replication origin firing and reducing the number of active forks, maintaining a stability of stalled replication forks, facilitating repair and promoting replication restart^24,25^. ATR maintains genome integrity by regulating the S/G2 transition, through inhibition of the CDK1-directed FOXM1 switch and inhibition of ATR results in premature activation of FOXM1 and inappropriate mitosis^26^. Another described function of ATR involves recruitment to the nuclear envelope in response to a mechanical stress during mitosis in dividing cells^27–29^. Here, the nuclear envelope linker of nucleoskeleton and cytoskeleton (LINC) complexes span the nuclear envelope and consist of conserved SUN domain and nesprin proteins which contribute to nuclear positioning and cellular rigidity during nucleokinesis^28,30^.

Cancer cells harbouring amplification of the *MYCN* gene and impaired ATM function, exhibit replication stress and cell cycle defects and are sensitive to ATR inhibitors^31,32^. Moreover, cancer cells with high levels of oncogene-driven replication stress rely on ATR activity for survival^33^. In recent years, a number of ATR inhibitors have been developed. These include M6620 and M4344 (Merck), AZD6738 (AstraZeneca) as well as BAY 1895344 (Bayer)^34,35^ that are currently at various phases in clinical trials (www.clinicaltrials.gov)^36^. ATR inhibition has not been extensively investigated in NB, although an earlier generation ATR inhibitor, VE-821, has been shown to synergise with PARP inhibitors to block NB cell growth^37^.

In previous comprehensive phosphoproteomics analyses of NB cells treated with ALK inhibitors, we noted changes in putative ATR target sites (S/TQ motifs) on the SAD1/UNC84 domain protein-2 (SUN2), a component of the LINC complex^38,39^. Here, we show that SUN2 is a target downstream of ALK and ATR in NB cells. Since cancer cells with high levels of oncogene-driven replication stress have been shown to rely on ATR activity for survival^23^, we hypothesised that ALK-driven NB cells would be sensitive to ATR inhibition. We show that the ATR inhibitor, BAY 1895344, is a potent inhibitor of NB cell growth. This robust effect of ATR inhibition by BAY 1895344 was confirmed in xenografts and two independent genetically engineered mouse models (GEMMs) of NB. Remarkably, a 14 day combined ATR/ALK inhibition therapeutic protocol completely ablated tumours in all ALK-driven-NB GEMMs treated. Phosphoproteomics and RNA-Seq analyses identified a strong E2F response in treated cells and tumours, as well as key targets of ATR activity in response to replication stress in NB cells (including FANCD2, FANCI, ATRX and DCK). The rapid tumour shrinkage in response to ATR inhibition with BAY 1895344 was accompanied by an inflammatory signature and extensive immune infiltration of tumours, implying that ATR inhibition does not negatively impair the immune response. Taken together, these results strongly suggest that inhibition of ATR could be of therapeutic value in NB, and highly motivates further exploration in this context.

## Results

### Phosphoproteomics identify ATR as a target of ALK signalling in NB cells

**Fig. 1:**
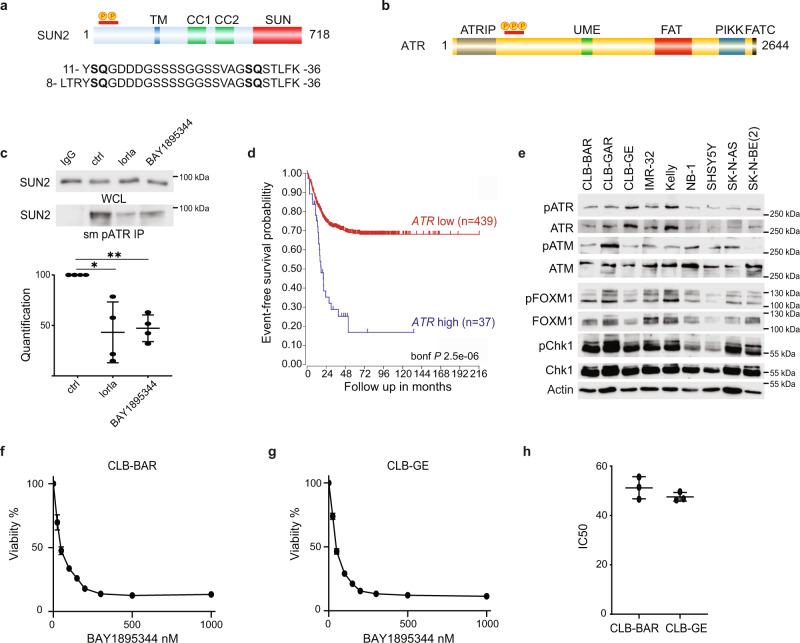
ATR signalling is required for survival in ALK-driven NB cell lines. **a** Graphic representation of SUN2 protein with localisation of the phosphorylation sites regulated by ALK signalling activity. SUN2 contains transmembrane domain (TM, blue), two coiled-coil domains (CC1 and CC2, green) and SUN (red) domains. The amino acid sequence of the two independent phosphopeptides identified by MS/MS is shown below with *SQ ATR phosphorylation motifs highlighted in bold. **b** Graphic representation of ATR protein structure with localisation of phosphorylation sites (S435, S436 and S437) regulated by ALK signalling activity. ATR contains HEAT repeats, an ATRIP binding (ATRIP) domain (grey), a nuclear localisation sequence, a UVSB PI3 kinase, MEI-41 and ESR1 (UME) domain (green), a FRAP-ATM-TRRAP (FAT) domain (red), a phosphatidylinositol-3 kinase-related protein kinase (PIKK) domain (blue) and a FRAP-ATM-TRRAP-C-terminal (FATC) domain (black). **c** Immunoprecipitation using ATR/ATM phospho-substrate motif antibodies in the presence or absence of ALK (lorlatinib) or ATR inhibitors (BAY 1895344) as indicated, followed by immunoblotting for SUN2 (WCL, whole-cell lysate; IP, immunoprecipitation) in CLB-BAR cells. Quantification of pATR immunoprecipitated SUN2 signal to total SUN2 signal is shown below. Data are presented as mean ± SD. *n* = 4 biologically independent experiments. *p = 0.032; **p = 0.0054, Student’s paired *t*-test, two-tailed distribution. **d** Kaplan-Meier event-free survival curves of 476 patients with NB from the Kocak cohort stratified according to ATR expression (https://r2.amc.nl). Patients with higher expression are highlighted in blue, whereas patients with lower expression are highlighted in red (*p* *Bonferroni-corrected* = 2.5 × 10^−6^). **e** Immunoblotting of whole-cell lysates from nine NB cell lines (CLB-BAR, CLB-GAR, CLB-GE, IMR-32, Kelly, NB-1, SHSY5Y, SK-N-AS and SK-N-BE(2)) probed with anti-: pATR, ATR, pATM, ATM, pFOXM1, FOXM1, pCHK1, CHK1 and actin antibodies. *n* = 3 biologically independent experiments. Cell lines are described in detail in Supplementary Table 1. **f** CLB-BAR cell viability in response to increasing concentrations of BAY 1895344. Data are presented as mean ± SD. *n* = 3 biologically independent experiments. **g** CLB-GE cell viability in response to increasing concentrations of BAY 1895344. Data are presented as mean ± SD. *n* = 3 biologically independent experiments. **h** IC50 values for CLB-BAR (51.24 ± 3.66 nM) and CLB-GE (47.57 ± 1.44 nM) cell lines calculated for BAY 1895344 from (**f**) and (**g**). Data are presented as mean ± SD. Source data are provided as a Source Data file.

We have previously published an analysis of ALK TKI treatment in NB cells describing changes in the phosphoproteome^39^. One of the most prominent hits in these datasets was the Sad1 and UNC84 Domain Containing 2 (SUN2) protein, which exhibited extensive changes in serine/threonine phosphorylation on an N-terminal peptide (corresponding to residues 8–36) in response to ALK inhibition (Fig. 1a)^38,39^. Phosphorylation of serine 12 in SUN2 was reduced in multiple NB cells lines on ALK TKI treatment (log2FC −2.76 in CLB-BAR; log2FC −2.11 in CLB-GE cells treated with crizoitinib)^39^. Interestingly, SUN2 phosphorylation has been identified in a large number of studies employing anti-ATR/ATM substrate antibodies that detect phosphorylated peptides containing the *S/TQ- ATR/ATM phosphorylation motif (www.phosphositeplus.org). Because this raises the possibility that ALK activity in NB cells modulates ATR/ATM activity, we examined ATR and ATM in our phosphoproteomic dataset, identifying serines 435, 436 and 437 on ATR (Fig. 1b) as sites dephosphorylated in response to ALK inhibition (log2FC −2.66 in CLB-BAR; log2FC −1.98 in CLB-GE cells treated with crizoitinib)^39^. Interestingly, serine 435 has been described as a PKA-mediated ATR site that regulates ATR activity^40^, and its phosphorylation is decreased in NB cells treated with ALK inhibitors^39^.

We validated these observations in CLB-BAR NB cells, where pATR/ATM substrate motif antibodies were able to immunoprecipitate SUN2 protein. In the presence of either the ALK TKI lorlatinib or the ATR inhibitor BAY 1895344, the levels of SUN2 protein immunoprecipitated by pATR/ATM substrate motif antibodies were reduced (Fig. 1c). These initial observations suggested that ATR may be active in NB cells, motivating its further exploration as a therapeutic target in NB.

### ALK-driven NB cell lines are sensitive to ATR inhibition

Investigation of an earlier published NB cohort (Kocak, 649 patients^41^) showed that increased ATR expression is associated with poor prognosis in NB (*P*_*adj*_ = 2.5 × 10^−6^; Fig. 1d). A panel of nine NB cell lines (CLB-BAR, CLB-GAR, CLB-GE, IMR-32, Kelly, NB-1, SHSY5Y, SK-N-AS, SK-N-BE(2)), which represent a range of aberrant genetic backgrounds found in NB tumours (Supplementary Table 1), were examined for the presence of ATR/pATR and the related PIKK ATM/pATM, and its downstream signalling components FOXM1/pFOXM1 as well as CHK1/pCHK1. All NB cell lines tested expressed ATR and detectable pATR, pATM, pFOXM1 and pCHK1 (Fig. 1e). This suggests that these cell lines have a basal level of DDR pathway activity. A number of ATR inhibitors have been developed, including BAY 1895344, which has recently been reported^34^. To further explore ATR as a therapeutic target in NB, we tested the effect of BAY 1895344 on CLB-BAR (*MYCN* amplified, *ALK*^*Δexon4–11*^) and CLB-GE (*MYCN* amplified, *ALK*^*F1174V*^) NB cells (Fig. 1f, g). Viability of both cell lines was highly sensitive to BAY 1895344 treatment displaying IC50s of 51.24 ± 3.66 and 47.57 ± 1.44 nM, respectively (Fig. 1h).

### ATR inhibition blocks NB cell growth regardless of ALK or MYCN status

**Fig. 2:**
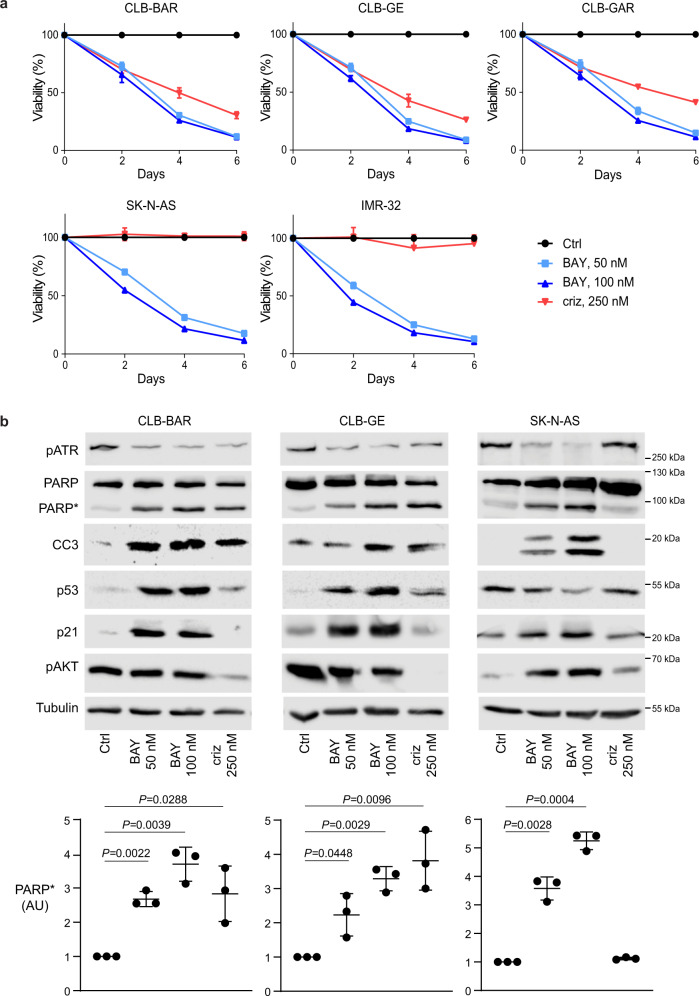
Effect of BAY 1895344 on NB cells. **a** Viability of NB cells treated with either 250 nM crizotinib (criz), 50 or 100 nM BAY 1895344 (BAY) as indicated. NB cell viability was measured after 2, 4 and 6 days for five different cell lines. ALK-addicted cell lines: CLB-BAR, CLB-GE, CLB-GAR; ALK non-addicted cell lines: SK-N-AS and IMR-32 (cell lines described in detail in Supplementary Table 1). **b** Apoptosis was monitored by immunoblotting for PARP cleavage (PARP*, quantified below), cleaved caspase 3 (CC3), p53, p21 and tubulin (as loading control) in NB cell lines after 24 h treatment with BAY 1895433 (50 or 100 nM). pAKT was employed as readout of ALK inhibition. 250 nM crizotinib was employed as positive control in CLB-BAR and CLB-GE cells. Data are presented as mean ± SEM. *n* = 3 biologically independent experiments. Student’s paired *t*-test, *p* values indicated. Source data are provided as a Source Data file.

Analysis of ATR activity identified pATR expression across a broad panel of NB cell lines, including non-ALK-driven cell lines (Fig. 1e). We therefore investigated the effect of BAY 1895344 on a range of NB cells, including those not driven by ALK. NB cells (CLB-BAR, CLB-GE, CLB-GAR, SK-N-AS and IMR-32) were treated with either 50 or 100 nM BAY 1895344 or 250 nM crizotinib and proliferation monitored over 6 days (Fig. 2a). Remarkably, all NB cell lines tested were sensitive to inhibition with both 50 and 100 nM BAY 1895344. As expected, the ALK-driven CLB-BAR, CLB-GE and CLB-GAR cell lines were also sensitive to 250 nM crizotinib, while proliferation of SK-N-AS and IMR-32 cells that are not ALK-driven was unaffected by crizotinib (Fig. 2a). To confirm the ATM activity in these NB cell lines, we treated ALK-addicted CLB-BAR and CLB-GE NB cells with the DNA damage-inducing agents etoposide and teniposide and examined activation of ATM. As expected, we observed increased cleaved PARP, p53, pATM and pATR levels in response to DNA damage-inducing agents (Supplementary Fig. 1).

The cellular effects of BAY 1895344 were monitored employing PARP cleavage as readout. CLB-BAR, CLB-GE and SK-N-AS NB cell lines were treated with either BAY 1895344 (50 or 100 nM) or crizotinib (250 nM) and cell lysates immunoblotted for pATR, PARP/cleaved PARP (PARP*), cleaved caspase 3 (CC3), p53, p21, pAKT and tubulin as loading control (Fig. 2b). In agreement with previous findings, the ALK-addicted CLB-BAR and CLB-GE cell lines undergo apoptosis in response to addition of crizotinib, resulting in increased levels of cleaved PARP^42^ and cleaved caspase 3. A similar robust increase in cleaved PARP was also observed in CLB-BAR and CLB-GE cells treated with either 50 or 100 nM BAY 1895344. Additionally, we also observed increases in cleaved caspase 3 and p53 levels upon treatment of cells with either 50 or 100 nM BAY 1895344 (Fig. 2b). SK-N-AS cells did not exhibit a detectable increase in PARP cleavage in response to crizotinib, but did show increased cleaved PARP levels in response to BAY 1895344 treatment.

In addition to pharmacological inhibition of ATR, we employed siRNA to knock down ATR in CLB-BAR cells, confirming that loss of ATR reduced cell proliferation with a concomitant increase in cleaved PARP in siRNA-transfected cells (Supplementary Fig. 2). Taken together, these results suggest that a wide range of NB cells are sensitive to ATR inhibition, exhibiting an apoptotic response and reduced proliferation.

### Transcriptional and proteomic profiling of ATR inhibition in NB cells

**Fig. 3:**
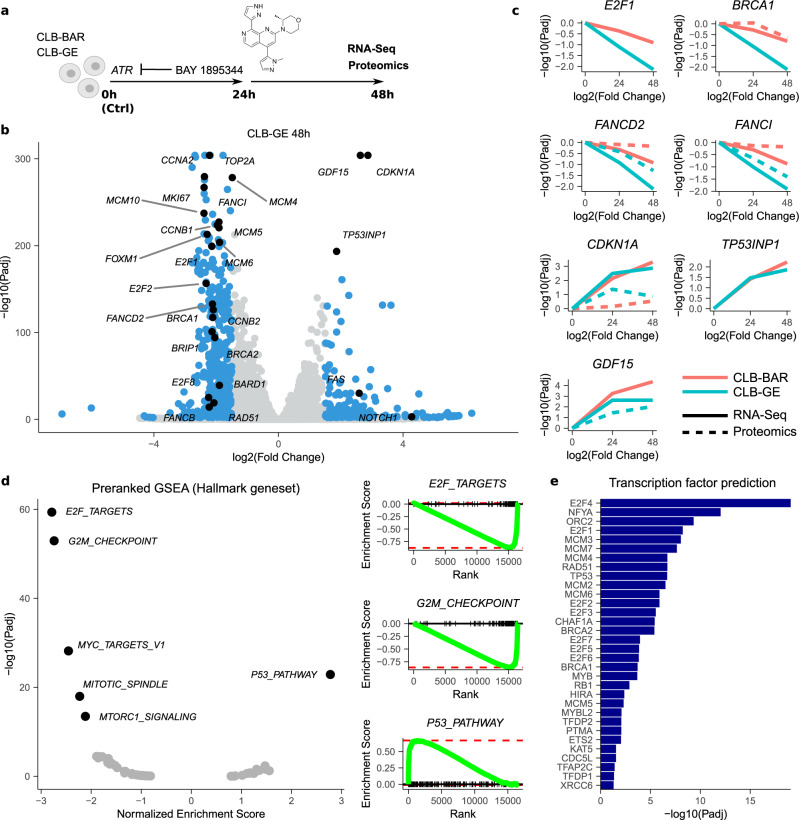
Transcriptomic and proteomic response to BAY 1895344 treatment of NB cells. **a** CLB-BAR and CLB-GE NB cells were treated for 24 or 48 h with 50 nM BAY 1895344 and differential gene and protein expression (DE) with untreated control conditions was determined using RNA-Seq or proteomics. See Supplementary Dataset 1 for the detailed results. **b** Volcano plot showing RNA-Seq DE response (log2FoldChange ±1.5 at 1% FDR) in CLB-GE cells 48 h post treatment. DE genes indicated in blue with genes discussed in the main text indicated and labelled in black. **c** Differential gene (full line) and protein (dashed line) expression dynamics after BAY 1895344 treatment for different genes as indicated. Response in CLB-BAR and CLB-GE cells indicated in red and green, respectively. **d** Preranked Hallmark GSEA results after ranking of genes following the DESeq2 statistic (CLB-GE response 48 h). Left panel shows normalised enrichment scores and corresponding FDR values with labelling of the most enriched gene sets. Right panels represent running score plots for the most enriched gene sets as indicated. **e** Transcription factor prediction based on a transcription factor target GSEA. GSEA was performed using Fisher’s exact test. Bars represent adjusted *p* values (−log scale) for enriched transcription factors at 5% FDR. Source data are provided as a Source Data file.

To characterise the downstream gene expression response following ATR inhibition, we performed a comprehensive RNA-Seq analysis after treatment of CLB-BAR and CLB-GE NB cells with 50 nM BAY 1895344 for 24 and 48 h (Fig. 3a). The strongest differential expression (DE) with untreated control cells was observed in CLB-GE cells after 48 h of treatment, with a downregulation of 370 genes and upregulation of 198 genes (log2 fold change threshold 1.5 at 1% false discovery rate (FDR); Fig. 3b). The downregulation was slower than the upregulation response (3 of 370 downregulated genes significant after 24 h, versus 70 (35%) of 198 upregulated genes; Supplementary Fig. 3) and included both *BRCA* genes, the *BRCA* interactors *BRIP1* and *BARD1*, *RAD51*, several E2F transcription factors (*E2F1*, *E2F2*, *E2F8*), *TOP2A*, *MKI67*, cyclins (e.g. *CCNA2* and *CCNB1/2*), the cyclin-regulating gene *FOXM1, MCM4/5/6/10* and genes from the Fanconi anaemia complementation group (*FANCD2*, *FANCB*, *FANCI*; Fig. 3b, c and Supplementary Dataset 1). These downregulated genes were strongly enriched for cell cycle-related targets of E2F transcription factors (*Padj* = 3.1e-59) and G2/M checkpoints (*Padj* = 7.4e-53), as observed from a preranked Gene Set Enrichment Analysis (GSEA) using a set of Hallmark genes^43^ (Fig. 3d). The upregulated genes included *CDKN1A*, *GDF15*, *FAS*, *NOTCH1* and *TP53INP1* and were mainly enriched for p53 pathways (*Padj* = 1.3e-23). A proteomics analysis indicated a highly similar expression response and enrichment results at the protein level (Fig. 3c, Supplementary Fig. 4 and Supplementary Dataset 1) and a GSEA-based transcription factor prediction confirmed a crucial role for E2F transcription factors, as well as several transcription factors known to be involved in DDR, such as RAD51 and BRCA1/2 (Fig. 3e).

### ATR regulates the S/G2 checkpoint in NB cells

**Fig. 4:**
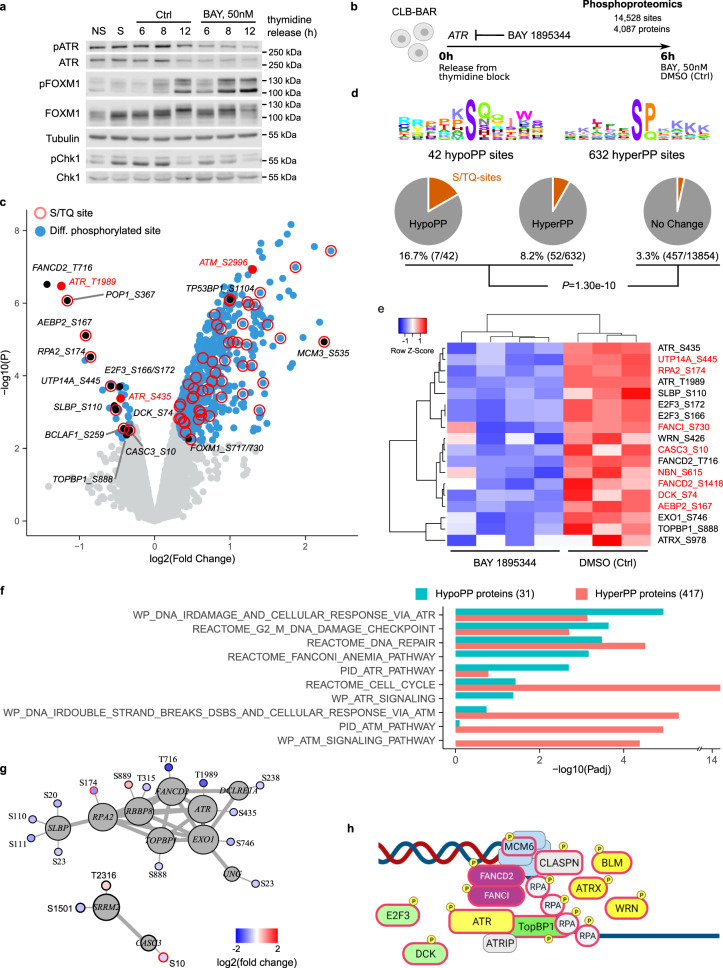
Phosphoproteomic response to BAY 1895344 treatment of NB cells. CLB-BAR NB cells were synchronised by thymidine block and treated with either DMSO (Ctrl) or 50 nM BAY 1895344. **a** Immunoblotting with anti pATR, ATR, pFOXM1, FOXM1, pCHK1, CHK1 and tubulin antibodies after treatment for 6–12 h as indicated. NS, non-synchronised; S, synchronised. **b**–**g** Phosphoproteomic analysis. **b** Differential phosphorylation (DP) upon 6 h BAY 1895344 treatment was determined by comparing the phosphoproteomic response with control (DMSO) conditions. See Supplementary Dataset 1 for the detailed results. **c** Volcano plot showing DP response. DP sites indicated in blue. ATR and ATM sites indicated and labelled in red. Other sites that are discussed in the main text in black. S/TQ sites indicated by red borders. **d** Linear motif analysis. Sequence logo plots showing position-specific enrichment ±5 amino acids centred around the phosphorylation site for hypo- and hyperphosphorylated sites as indicated. Pie charts showing the proportion of S/TQ motifs in hypophosphorylated, hyperphosphorylated and non-DP sites. *p* Value calculated using Fisher’s exact test, comparing S/TQ sites in DP versus non-DP sites. **e** Heatmap comparing *z*-score normalised phosphorylation signals between control (DMSO) and BAY 1895344 conditions for hypophosphorylated sites that are discussed in the main text. S/TQ sites labelled in red. **f** GSEA on 31 proteins with hypophosphorylated sites and 417 proteins with hyperphosphorylated sites. Bar plot showing a selection of Canonical pathways that were enriched at 5% FDR (*q* < 0.05). See Supplementary Dataset 2 for the detailed results. **g** STRING network analysis. Subnetworks showing all 1st-degree interacting proteins with hypophosphorylated sites. Log2 (fold change) values labelled by heatscale (key indicated on bottom right). S/TQ sites indicated by red borders. **h** Schematic highlighting key targets of ATR activity in NB cells. Source data are provided as a Source Data file.

Our transcriptomic/proteomics results are in line with ATR regulation of an S/G2 checkpoint that controls mitotic entry^26^. To confirm that this checkpoint is indeed under control of ATR in NB cells, we monitored downstream ATR targets pFOXM1 (T600) and pChk1 (S345) levels during the cell cycle. CLB-BAR cells were synchronised by thymidine block and levels of pFOXM1 and pCHK1 monitored in the presence or absence of BAY 1895344 for 6, 8 and 12 h after thymidine release (Fig. 4a). We observed enhanced pFOXM1 present at earlier time points in CLB-BAR cells released from thymidine block in response to ATR inhibition by BAY 1895344. Similar regulation of pFOXM1 and pCHK1 was observed in synchronised CLB-GE cells released from thymidine block in the presence of BAY 1895344 (Supplementary Fig. 5a). We also tested whether inhibition of ALK, with lorlatinib, had any effect on the ability of cells to pass the S/G2 checkpoint. In contrast to ATR inhibition, ALK inhibitors had no effect on pFOXM1 (Supplementary Fig. 5b). Thus, regulation of the S/G2 checkpoint by ATR is conserved in NB cells.

### ATR activity regulates phosphorylation of key DDR components in NB cells

To further investigate the effect of BAY 1895344 on NB cells, we performed a phosphoproteomic analysis of CLB-BAR cells treated with 50 nM BAY 1895344 for 6 h after release from thymidine block (Fig. 4b). In all, 14,528 phosphosites (12,792 serine, 1679 threonine and 57 tyrosine) were identified in 4087 different proteins. 674 sites in 444 different proteins were differentially phosphorylated in response to BAY 1895344 treatment (log2 fold change threshold 0.3 at 5% FDR; Fig. 4c and Supplementary Dataset 1).

As expected, ATR was dephosphorylated at both the T1989 autophosphorylation site and on S435 (log2FC = −1.24 and −0.45, respectively). ATR-specific S/TQ motifs were 2.7 times more frequent in the sites with altered phosphorylation signals as compared to the non-responding sites (8.8% versus 3.3%; *P* = 1.3e-10, Fisher’s exact test; Fig. 4d), with 7 of 42 hypophosphorylated sites containing an S/TQ motif (16.7%, as compared to 8.2% of the 632 hyperphosphorylated sites). These hypophosphorylated S/TQ sites included AEBP2 S167 (−0.92), UTP14A S445 (−0.58), DCK S74 (−0.52) and CASC3 S10 (−0.33) (Fig. 4c, e). Non-S/TQ sites included the ATR substrate FANCD2 T716 (−1.43), E2F3 S172/166 (−0.47), SLBP S110 (−0.54) and TOPBP1 S888 (−0.37). A GSEA indicated that proteins containing these hypophosphorylated sites were significantly enriched for DNA repair (*Padj* = 3.3e-04) and related pathways, such as G2M checkpoints (*Padj* = 2.3e-04) and the Fanconi anaemia pathway (*Padj* = 6.8e-04; Fig. 4f). The involvement of these proteins in DDR pathways was further confirmed from their enrichment for an earlier annotated set of 276 DDR genes (25.8% of hypophosphorylated proteins identified as DDR genes versus 3.1% of other proteins, *P* = 4.5e-06)^44^. Further, most proteins responsible for these enrichments are known as directly interacting proteins, as observed from a STRING network analysis (Fig. 4g). In this regard, it is noteworthy that weaker hypophosphorylation signals were also observed in several other interacting and DDR-related proteins, such as ATRX, FANCI, EXO1 and WRN (Fig. 4e).

Our results show that the majority of hypophosphorylated sites do not contain S/TQ motifs, suggesting other, indirect modulations. These secondary modulations could also explain the strong hyperphosphorylation response (632 sites in 417 proteins) that was observed upon BAY 1895344 treatment (Fig. 4c). This response included FOXM1 S717/730 (log2FC= 0.45), the PIKK family member ATM S2996 (1.30)^45^, and a number of ATM/ATR S/TQ target phosphorylation sites, such as MCM3 S535 (2.25) and TP53BP1 S1104 (1.00). These results are in line with a compensatory activation of ATM, as suggested by the strong enrichments for ATM-related pathways in the proteins containing hyperphosphorylated sites (Fig. 4f) and in line with earlier findings suggesting that ATM is not inhibited by BAY 1895344^34^. In summary, our phosphoproteomics analysis identified a number of ATR targets in NB cells, including components of the DNA repair machinery that are critical to manage oncogene-induced replication stress, as well as E2F3 and DCK as important ATR targets (Fig. 4h).

### BAY 1895344 inhibits proliferation and induces cell death of NB cells

**Fig. 5:**
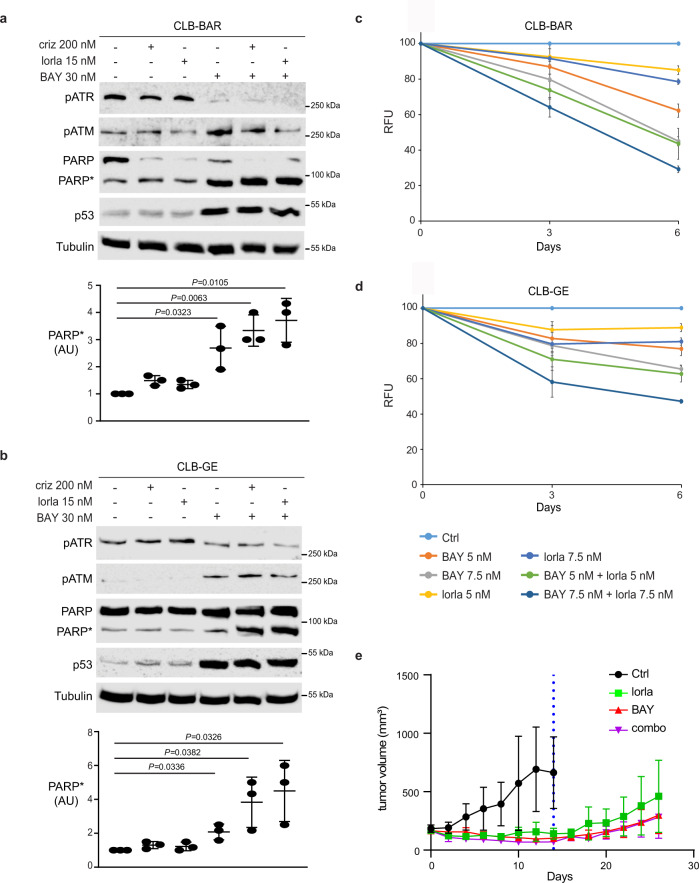
BAY 1895344 treatment inhibits growth of NB xenografts. **a, b** ALK-positive neuroblastoma cell lines CLB-BAR (**a**) and CLB-GE (**b**) were treated with crizotinib (criz), lorlatinib (lorla) or BAY 1895344 (BAY), either alone or in combination, as indicated. Cell lysates were immunoblotted for pATR, pATM, PARP/cleaved PARP (PARP*) and p53. Tubulin was used as a loading control. *n* = 3 biologically independent experiments. Student’s paired *t*-test, *p* values indicated. **c, d** Resazurin assay-based viability in NB cell lines CLB-BAR (**c**) and CLB-GE (**d**) treated with BAY 1895344 or lorlatinib alone or in combination, as indicated. Data are presented as mean ± SEM relative to untreated cells. *n* = 3 biologically independent experiments. **e** Female *BALB/cAnNRj-Foxn1nu* mice were treated with lorlatinib (10 mg/kg twice daily, *n* = 3), BAY 1895344 (50 mg/kg twice daily; with a 3 day on/4 day off cycle, *n* = 5) or as combination (*n* = 3) for 14 days (dotted line). Animals in each treated group were maintained for a further 12 days after treatment ended (14–26 days). Data are presented as mean tumour volumes (mm^3^) ±SD. One-way ANOVA followed by Sidak´s multiple comparisons test was used to calculate significant differences between groups. *p* = 0.003 for Ctrl v lorlatinib, *p* < 0.0001 for Ctrl v BAY 1895344 and Ctrl v combination. Source data are provided as a Source Data file.

We, and others, have previously shown that the third generation ALK TKI lorlatinib abrogates growth of ALK-driven NB cell lines^46,47^. Lorlatinib is now included in several clinical trials for NB driven by ALK activating mutations as well as ALK amplification (https://clinicaltrials.gov/). To address whether inhibiting ALK, ATR or a combination of both would affect cell proliferation and cell death, ALK-addicted NB cells were treated either with ALK inhibitors (crizotinib or lorlatinib) and/or BAY 1895344. We observed increased cleaved PARP expression when ALK inhibitors were employed in combination with low concentrations of ATR inhibitor (30 nM) when compared to the single agents alone (Fig. 5a, b). In agreement with our findings from the analysis of the ATRi phosphoproteome, we also observed an increased phosphorylation of ATM upon treatment with BAY 1895344 (Fig. 5a, b). We next investigated whether ALK inhibition with lorlatinib treatment could synergise with BAY 1895344 to impair growth of ALK-driven NB cell lines, such as CLB-BAR and CLB-GE. ALK-addicted NB cell lines were treated with either BAY 1895344 (5 or 7.5 nM) or lorlatinib (5 or 7.5 nM), alone or in combination, for 3 and 6 days (Fig. 5c, d). Combination treatment employing lorlatinib together with BAY 1895344 showed mild synergy when compared to treatment with lorlatinib or BAY 1895344 alone in CLB-BAR and CLB-GE cell lines (Fig. 5c, d). Single-agent treatments reduced proliferation by 10–30% by day 6, whereas a combinatorial treatment of BAY 1895344 and lorlatinib showed a further decrease in proliferation (Fig. 5c, d). Combination indexes (CI) were calculated according to the Chou and Talalay method^48^, indicating mild synergism (for CLB-BAR: CI = 0.85, BAY 1895344 5 nM/lorlatinib 5 nM; CI = 0.90, BAY 1895344 7.5 nM/lorlatinib 7.5 nM and for CLB-GE: CI = 0.89, BAY 1895344 5 nM/lorlatinib 5 nM; CI = 0.92, BAY 1895344 7.5 nM/lorlatinib 7.5 nM).

### BAY 1895344 inhibits tumour growth in a xenograft NB model

Having characterised BAY 1895344 and lorlatinib in NB cells we next tested their ability to block growth of NB in mouse xenografts. Subcutaneous injection of CLB-BAR NB cells led to tumours that were treated with BAY 1895344 (*n* = 5), lorlatinib (*n* = 3), or a combination of BAY 1895344 and lorlatinib (*n* = 3). Lorlatinib (10 mg/kg) was delivered twice daily, while BAY 1895344 (50 mg/kg) was delivered twice daily with a 3 day on/4 day off schedule based on previous studies^34^. Tumour growth inhibition (TGI) values of 117.6%, 138.7% and 156.9% were observed with lorlatinib, BAY 1895344 and the combination, respectively (Fig. 5e). Tumours in the vehicle control group continued to grow reaching a significant increase compared to lorlatinib, BAY 1895344 or combination treatment after day 6 (*p* = 0.0122) (Fig. 5e). As expected, lorlatinib displayed antitumour activity in agreement with previous reports^46^. Tumour volume was significantly decreased at day 14 in all treatment groups (Fig. 5e). Upon treatment cessation at 14 day tumour growth resumed, with animals treated with BAY 1895344 showing a somewhat slower, but nonsignificant, take-off than those treated with lorlatinib alone.

### BAY 1895344 eliminates tumours in two independent Alk-driven NB GEMMs

**Fig. 6:**
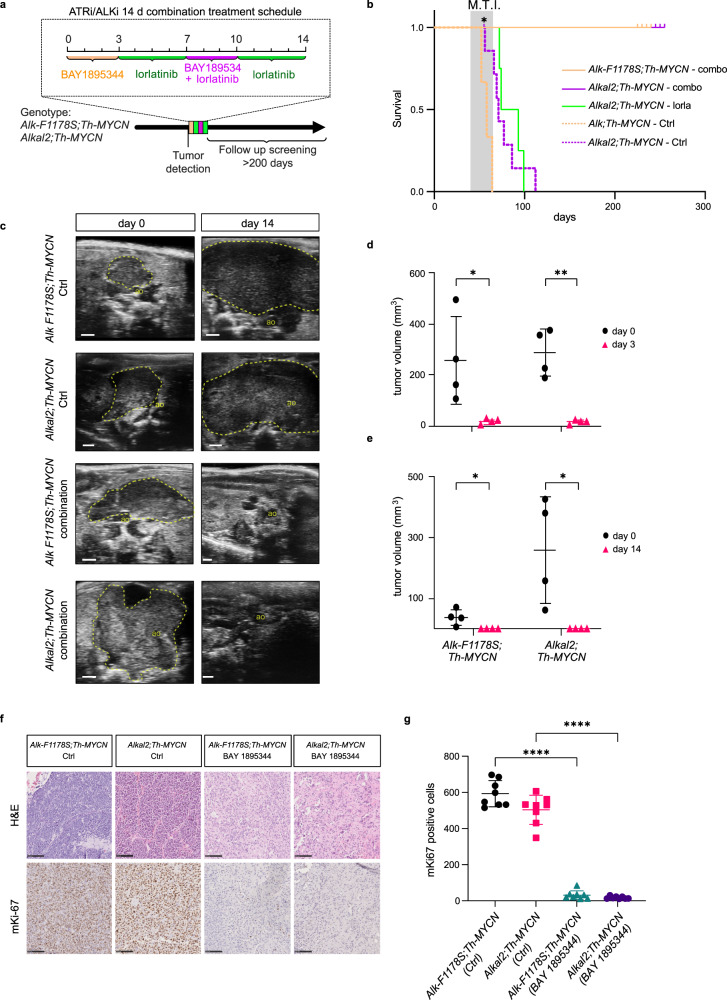
BAY 1895344 treatment cures *Alk/MYCN*-driven NB in GEMMs. **a** Schematic overview of treatment regimen for *Alk/MYCN*-driven GEMM tumours, consisting of 25 mg/kg BAY 1895355 for 3 days, followed by 10 mg/kg lorlatinib for 11 days, supplemented with BAY 1895344 at days 8–10, orally. All treatments were given twice daily. **b** Survival (from birth) in *Alk-F1178S;Th-MYCN* (tan, *n* = 4), *Rosa26_Alkal2;Th-MYCN* (magenta, *n* = 4) mice compared with mice treated continuously for 30 days with lorlatinib 10 mg/kg b.i.d. from tumour detection (green, *n* = 4) and *Alk-F1178S;Th-MYCN* (tan dashed, *n* = 3), *Rosa26_Alkal2;Th-MYCN* (magenta dashed, *n* = 7) that received no treatment. Shadowed areas represent mean tumour incidence (±SD) in the treatment group. *n* ≥ 4 for each treatment group. *Indicates one *Rosa26_Alkal2;Th-MYCN* animal that exhibited a complete response sacrificed after treatment. M.T.I., mean tumour incidence. **c** Representative ultrasound images from *Alk-F1178S;Th-MYCN* and *Rosa26_Alkal2;Th-MYCN* animals treated for 14 days with either combination or vehicle control. Abdominal aorta (ao), tumour border (dashed line). Scale bar is 1 mm. **d** Tumour volume (mm^3^) change in mice treated 25 mg/kg b.i.d. BAY 1895344 for 3 days. Data are presented as mean ± SD. *n* = 4 per genotype. *p = 0.0331, **p = 0.0057, one-tailed paired *t*-test. **e** Tumour volumes (mm^3^) at start and end of combination treatment. Data are presented as mean ± SD. *n* = 4 per genotype, *p = 0.0292 for *Alk-F1178S;Th-MYCN*, *p = 0.0301 for *Rosa26_Alkal2;Th-MYCN*, one-tailed paired *t*-test. **f** Representative immunohistochemical mKi67 staining for tumours of indicated genotype treated for 3 days with either BAY 1895344 25 mg/kg b.i.d. or vehicle control (scale bar 100 µm), quantified in (**g**). Data are presented as mean number of Ki67-positive cells ±SD in 8 representative fields of view, *n* = 4 biologically independent samples, ****p < 0.0001, one-way ANOVA with Sidak corrections for multiple comparisons. Source data are provided as a Source Data file.

The BAY 1895344 ATRi has recently been characterised, and a phase I clinical trial in solid tumours in adults has been conducted^34,35^. While we observed only mild synergy when combining ATR and ALK inhibition, we rationalised that combination with lorlatinib, which has previously been shown to reduce tumour growth in ALK-driven NB mouse models, may improve treatment responses^46,49^. We therefore tested the efficacy of BAY 1895344 together with lorlatinib in two independent genetically modified mouse (GEMM) NB models—*Rosa26_Alkal2;Th-MYCN* (*n* = 4) and *Alk-F1178S;Th-MYCN* (*n* = 4)^49^. Tumours were treated with a 14 day regimen that combined BAY 1895344 together with the ALK TKI lorlatinib (3 days BAY 1895344 25 mg/kg b.i.d. (twice per day), 4 days lorlatinib 10 mg/kg b.i.d., 3 days combination, 4 days lorlatinib) (Fig. 6a). All mice tolerated the treatment regimen with no noticeable side effects. Remarkably, in contrast to vehicle control treated mice which exhibited aggressive tumour growth, no tumours were detected at day 14 after treatment in any of the treated GEMMs (Fig. 6b, c, e). One mouse of genotype *Rosa26_Alkal2;Th-MYCN* was sacrificed after 14 days of treatment (Fig. 6b, denoted with *), confirming no detectable tumour material on dissection. Lorlatinib monotherapy reduced tumour size relative to control at 14 days, but did not result in a complete resolution of tumour material (*Rosa26_Alkal2;Th-MYCN* (*n* = 4))^49^. Given this striking response to the 14 day BAY 1895344/lorlatinib regimen, we maintained all remaining mice over time without any therapeutic interventions monitoring regularly with ultrasound for tumour development. In addition, several mice were subjected to follow-up MRI scans at approximately day 50, confirming complete tumour regression. One mouse of genotype *Rosa26_Alkal2;Th-MYCN* was identified with an abdominal tumour (∅ > 14 mm) 100 days after initial treatment start and subjected to an additional 14 day regimen, exhibiting a second complete response (Supplementary Fig. 6). This same mouse relapsed again after 133 and 161 days, in both instances responding to treatment, before expiring at 196 days (Supplementary Fig. 6). Remarkably, all other mice have remained tumour free (for >200 days from treatment). Comparison of these results with treatment of NB mouse tumours in *Rosa26_Alkal2;Th-MYCN* animals with a 30 day lorlatinib treatment show a dramatic effect on survival when the 14 day BAY 1895344/lorlatinib combined regimen is employed (Fig. 6b).

### Gene expression analysis identifies a strong tumour response to BAY 1895344 treatment

To understand the underlying mechanism of this therapeutic intervention on NB tumour growth, we investigated gene expression in treated tumours from both *Rosa26_Alkal2;Th-MYCN* and *Alk-F1178S;Th-MYCN* relative to untreated controls. To achieve this, BAY 1895344 was administered in a short time course of 3 days (25 mg/kg twice daily) in a second cohort of mice (*Rosa26_Alkal2;Th-MYCN* and *Alk-F1178S;Th-MYCN*, treated with either BAY 1895344 or vehicle control; *n* = 4 per treatment group) and gene expression analysed by RNA-Seq. Analysis of tumour size by ultrasound confirmed a robust tumour shrinkage in response to BAY 1895344 treatment at day 3 in both *Rosa26_Alkal2;Th-MYCN* and *Alk-F1178S;Th-MYCN* tumours (Fig. 6d). Tumours excised from mice after treatment with BAY 1895344 exhibited reduced staining for the proliferation marker Ki-67 when compared with vehicle treated controls (Fig. 6f, quantified in 6g).

**Fig. 7:**
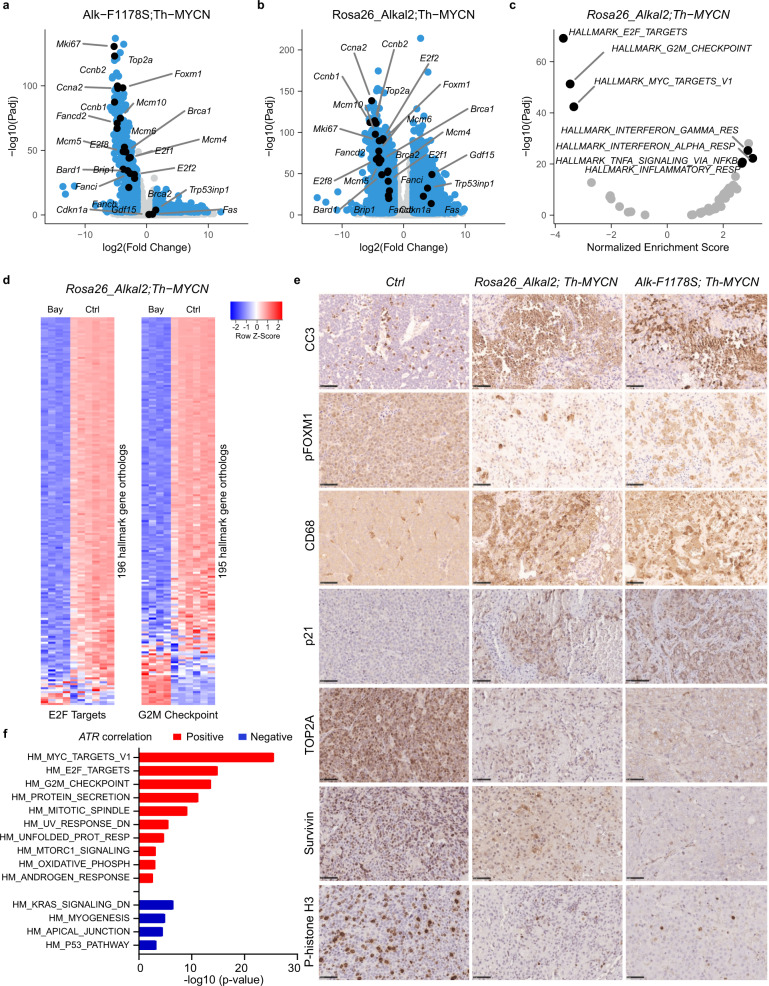
Transcriptomic response to BAY 1895344 treatment in *Alk/MYCN*-driven NB in GEMMs. RNA-Seq-based differential gene expression analysis between NB tumours from untreated control mice and mice treated with BAY 1895344 for 3 days. Two different mice models were used as indicated: *Alk-F1178S;Th-MYCN*; *Rosa26_Alkal2;Th-MYCN* (*n* = 4 treated per genotype, *n* = 6 controls). **a**, **b** Volcano plots with indication of DE genes in blue. DE thresholds are indicated by dashed lines. Orthologs of the genes indicated in Fig. 3b labelled. **c** Preranked Hallmark GSEA results after ranking of genes following the DESeq2 statistic. Plot shows normalised enrichment scores and corresponding FDR values with labelling of gene sets discussed in the main text. **d** Heatmap comparing *z*-score normalised gene expression counts between untreated control and BAY 1895344 treated *Rosa26_Alkal2;Th-MYCN* tumours for all genes from the Hallmark gene sets E2F Targets and G2M Checkpoints as indicated. Colour key at top right. **e** Histological examination of *Rosa26_Alkal2; Th-MYCN, Alk-F1178S;Th-MYCN* in response to BAY 1895344 treatment compared with vehicle control tumours. Staining for cleaved caspase 3 (CC3), pFOXM1, CD68, p21, TOP2A, Survivin and P-histone H3 is shown. Scale bars indicate 50 μm. *n* = 3 biologically independent samples. **f** Hallmark GSEA on genes with positive (red bars) or negative (blue bars) expression correlation to *ATR* gene expression in patient NB samples (correlation *Padj* < 0.01). Bar plot showing −log10 (*p* value) of Hallmark gene sets enriched with *Padj* < 0.01. Analysis and GSEA were performed on Kocak NB_649 tumour dataset (gse45547) using the R2: Genomics Analysis and Visualization Platform. Source data are provided as a Source Data file.

RNA-Seq analysis indicated a downregulation of 603 and 2220 genes in *Alk-F1178S;Th-MYCN* and *Rosa26_Alkal2;Th-MYCN* tumours, respectively (log2 fold change of 1.5 at 1% FDR; Fig. 7a, b and Supplementary Dataset 1). These genes were largely identical to the downregulation observed in NB cell lines, with similarly strong enrichments for E2F targets and G2M checkpoints (Fig. 7c, d and Supplementary Fig. 7). The specificity of the response to ATR inhibition is further highlighted by the striking correlation of cell line and mice differential expression log2 fold change values for these processes (Pearson correlation coefficients between 0.67 and 0.82; Supplementary Fig. 7). These mouse tumour transcriptomic responses are also in keeping with our phosphoproteomics analyses which identified E2F3 as a phosphorylation target of ATR activity (Fig. 4c). Interestingly, the TP53-related upregulation that was observed in NB cell lines was present in the *Rosa26_Alkal2;Th-MYCN* and not the *Alk-F1178S;Th-MYCN* tumours (Fig. 7a, b). Immunohistochemical analysis of tumours in *Rosa26_Alkal2;Th-MYCN* and *Alk-F1178S;Th-MYCN* mice confirmed a dramatic apoptotic response, as measured with cleaved caspase 3 (CC3), after 3 days of treatment (Fig. 7e). In addition, we used phosphohistone H3 as a marker for proliferation, observing that tumours treated with BAY 1895344 displayed decreased proliferation (Fig. 7e). As FOXM1 is a phosphorylation target of ATR, we also investigated pFOXM1 in treated tumours. Here we observed a remarkable change in subcellular localisation of pFOXM1 protein in BAY 1895344-treated tumours. pFOXM1 was excluded from the nucleus, and localised in the cytoplasm in treated tumours, in comparison with the almost exclusive nuclear localisation of pFOXM1 in control tumours (Fig. 7e). We also noted a strong enrichment for several immune-related processes (Fig. 7c). The rapid tumour shrinkage observed on ATR inhibition suggests a strong tumour and potentially host response that was identified as inflammatory signatures in our RNA-Seq analyses. Therefore, we tested whether host immune cell involvement may assist in the rapid tumour loss on ATR inhibition. Analysis of immune cells on treated tumours with anti-CD68 antibodies identified a remarkable infiltration of treated tumours in response to BAY 1895344 treatment, in comparison with vehicle treated controls (Fig. 7e). Thus, ATR inhibition with BAY 1895344 does not impact the function of the host immune system, but rather stimulates it. We also examined three additional markers that were significantly differentially expressed in our RNA-Seq analysis: p21 (encoded by *Cdkn1a;* 3.6 log2FC; p 2e-21), Top2A (*Top2a;* −4.6 log2FC; p 4.2e-88) and Survivin (*Birc5;* −4.6 log2FC; 6.6e-59). Expression of the G2/M checkpoint protein p21 increased in tumours treated with ATR inhibitor, while levels of both Top2A and the apoptosis inhibitor Survivin decreased, in agreement with RNA levels (Fig. 7e). Taken together, our immunohistochemical analyses show that BAY 1895344 induces a robust apoptotic response in the tumour together with a strong host immune response in *Alk/MYCN*-driven NB.

Interestingly, GSEA of genes positively or negatively correlating with ATR expression in human NB tumours (Kocak-649- GSE45547), using the R2: Genomics Analysis and Visualization Platform (http://r2.amc.nl), revealed a Hallmark gene set signature (Fig. 7f) that is nearly identical to the ATR inhibition response observed in either our NB cell lines (Fig. 3b–d) or mouse model RNA-Seq datasets (Fig. 7a–c). For instance, genes with positive correlation to ATR expression in NB tumours are similarly (Fig. 3b–d) and strongly enriched for cell cycle-related targets of E2F transcription factors (*Padj* = 1.30e-15) and G2/M checkpoints (*Padj* = 2.60e-14). In contrast, genes negatively correlating with ATR expression are enriched for other pathways including p53 pathway (*Padj* = 5.3e-4) (Fig. 7f). This further highlights and reinforces the specificity of the observed ATR inhibition gene expression response in our model systems.

## Discussion

Our initial findings of regulation of SUN2 phosphorylation downstream of ALK suggested a class of ALK signalling targets that may be involved in maintaining the integrity of the nuclear architecture. The observation of ATR/ATM PIKK phosphorylation motifs suggested a potential role for ATM or ATR and examination of publicly available phosphoproteomic data regarding SUN2 (including the www.phosphosite.org database) suggested that SUN2 phosphorylation has been frequently identified in anti-ATM/ATR substrate motif antibody-based experiments in cancer cells. Although phosphoproteomics data from peptides with multiple phosphorylation sites is challenging, many datasets report phosphorylation of the serine 12 (S*Q) site in SUN2 (www.phosphosite.org)^50^. ATR and ATM have similar substrate specificity, phosphorylating S/TQ motifs, and over 700 targets were described in a study of HEK293 cells^51^. This study also noted a connection between ATR/ATM substrates and signalling through the insulin receptor (INR), suggesting that the DDR might induce a survival signal through activation of AKT and that DDR signalling intersects with INR-PI3K-AKT signalling at several points (such as IRS, c-Raf, AKT, TSC1 and 4E-BP1)^51^. A comprehensive phosphoproteomics study in adipocytes also identified the ATR/ATM serine 12 (S*Q) site in SUN2 as a target of insulin signalling^52^. Moreover, it has been reported that IGF-1R signalling is required for optimal ATR-CHK1 kinase signalling in irradiated human keratinocytes^53^. These observations suggest that ALK, which is a member of the INR RTK superfamily, and ATR signalling, may be interdependent in NB cells. One hypothesis would be that ALK activity in NB cells ensures nuclear and genome integrity by supporting efficient ATR signalling, which may include maintaining effective phosphorylation of ATR substrates, such as SUN2. While the molecular effect of the SUN2 phosphorylation observed is unknown, the localisation of the observed phosphorylation sites are interesting. All are located in the amino-terminal portion of SUN2, which sits inside the inner nuclear envelope and is involved with binding nuclear lamin^54^. Interestingly, a study profiling UV-induced ATM/ATR signalling pathways identified the serine 12 (S*Q) site in SUN2 as well as lamin A/C as nuclear envelope protein targets of either ATM or ATR^55^. Modulation of these sites may result in altered SUN2–lamin interactions and subsequent modulation of nuclear structure, and is in line with the findings of the Foiani group, which has reported a role for ATR at the nuclear envelope in response to physical and chemical changes^29,30^. Interestingly, in a recent report, they identified another component of the LINC complex—Nesprin-2—as an ATR interactor, further supporting a role for ATR in maintenance of LINC-mediated nuclear-cytoskeleton architecture^29^.

Together, ATM and ATR are two of the most central kinases involved in the DDR, with ATM responding to double-strand breaks and ATR to single-strand breaks such as those found at sites of replication stress^23^. In NB, loss of chromosome 11q is a well-known marker of poor prognosis and is used in patient stratification^13,14,17^. In addition to loss of tumour suppressors such as *DLG2* or *SHANK2*, this region contains many genes involved in the DDR, such as *ATM*, *CHEK1*, *H2AFX* and *MRE11*, as noted previously by others^37^, raising the possibility that, e.g. 11q loss in NB may make NB more dependent on ATR in the response to oncogene-induced stress. In this study we investigated a panel of NB cell lines, including several with deletion of 11q, and were able to detect pATM, suggesting that there is ATM activity in these lines (Fig. 1). Other than 11q deletion, other high-risk NB groups are *MYCN*-amplification, *ALK*-GOF (gain of function), combined *MYCN*-amplification/*ALK*-GOF and 2p gain which may lead to high levels of oncogene-induced replicative stress and sensitivity to loss of ATR activity.

These observations led us to test ATR inhibitors on NB cells, initially focusing on ALK-driven NB cells (Figs. 1 and 2). Development of ATR inhibitors has been somewhat delayed due to concerns about the general importance of ATR, as knockout mice are embryonic lethal and ATR function is required in proliferating adult tissues^56–58^. However, several reports indicate that loss of ATR function renders cancer cells sensitive to oncogene-induced replication stress, e.g. in oncogenic RAS-driven tumours^33,59^. We hypothesised that ALK-driven NB, which often involves *MYCN* amplification, may also represent a situation where cancer cells are more dependent on the integrity of ATR-regulated checkpoint responses and therefore sensitive to ATR inhibitors. We tested two independent ATR inhibitors, AZD6738^60^ and BAY 1895344^34^, noting that the BAY 1895344 compound exhibited low IC50 values on both CLB-BAR and CLB-GE cell lines (Figs. 1 and 2).

Surprisingly, we found that all NB cell lines we tested were highly sensitive to ATR inhibition by BAY 1895344, even those lacking 11q deletion (CLB-BAR, CLB-GE and IMR-32 are not 11q del, in contrast to CLB-GAR and SK-N-AS). Furthermore, neither *Rosa26_Alkal2;Th-MYCN* nor *Alk-F1178S;Th-MYCN* NB mouse tumours harbour genetic abnormalities syntenic to human 11q deletions that would support increased sensitivity to ATRi treatment as a result of decreased ATM or other DDR response component activity^49^. A recent publication has explored the use of ATR inhibition with PARP inhibitors in NB cell lines^37^. This work employed VE-821 to inhibit ATR, and noted growth inhibition (GI) values of 0.66 to >20 μM, much higher than those observed in this study with BAY 1895344 (IC50 ~50 nM). Of note, VE-821 had no effect on growth of SK-N-AS cells (GI > 20)^37^, while we show here that 50 nM BAY 1895344 is highly effective in preventing growth of SK-N-AS NB cells. An additional report has suggested that *MYCN* amplified cell lines are more sensitive to ATR inhibition, in this case employing AZD6738, and demonstrated sensitivity in a *Th-MYCN*-driven mouse NB model^61^. Although we initially focused characterising ALK-driven NB cell lines, we found that all NB cell lines tested, including non-ALK-driven NB cells, were sensitive to inhibition of ATR with BAY 1895344, irrespective of either 11q or *MYCN* amplification status. We also explored a combination of ALK and ATR inhibition (employing lorlatinib and BAY 1895344 together), but could see only mild synergistic effects on proliferation of ALK-driven NB cells in vitro (Fig. 5). Thus, although our previous data indicate that ALK signalling regulates ATR phosphorylation on serine 435^39^, which has been reported as important for ATR activity^40^, we currently do not fully understand the potential advantages of targeting them in combination.

Using BAY 1895344 we were also able to confirm that the regulation of FOXM1 at the S/G2 checkpoint by ATR is conserved in NB cells^26^ (Fig. 4). A number of studies have examined phosphorylation in response to ATR inhibition^51,55^, but to our knowledge, none has been reported with BAY 1895344. Here we report the first phosphoproteomics analysis of BAY 1895344. While the identification of specific phosphorylation signals in proteins with multiple phosphorylation sites is challenging, we identified a number of proteins that are specifically phosphorylated. Among these were a number of proteins phosphorylated on the PIKK S/TQ substrate motif^21^, in NB cells. We find that BAY 1895344 effectively inhibits ATR but not ATM^34^ in NB cells, and observe upregulation of ATM phosphorylation on S2996, which is a previously reported ATM autophosphorylation site^45^, together with a number of downstream substrates in our phosphoproteomics analysis suggesting that addition of BAY 1895344, while inhibiting ATR, results in an upregulation of ATM presumably as a compensatory measure (Fig. 4). In agreement with this finding, a recent interesting report used phosphoproteomics to investigate the relative contribution of the different PIKKs, highlighting a dynamic relationship of activity between ATR and ATM^62^. In addition to phosphorylation of critical components of the DDR response, including FANCD2 and FANCI, on ATR inhibition with BAY 1895344, we were interested to note that ATRX, E2F and DCK are targets of ATR activity in NB cells. *ATRX* mutation or deletion represents a well-known NB subgroup^6^. The identification of ATRX as an ATR target in NB cells is in line with a recent finding of ATRX in complex with FANCD2 at common fragile sites in the genome, and where ATRX plays a role in stability at these sites^63^. Deoxycytidine kinase (DCK) is a rate-limiting enzyme in the nucleoside salvage pathway, and phosphorylation of DCK (S74) by ATR has been shown to inhibit its activity^64,65^, reducing nucleoside availability through the salvage pathway, potentially providing additional problems for the tumour cell to overcome. Why the related PIKK ATM, which is apparently activated in response to ATR inhibition in NB cells, is unable to compensate for ATR in the case of DCK, is currently unclear. Interestingly, Schlam-Babayov and colleagues noted a dynamic relationship of activity between ATR and ATM, with differences in their ability to compensate for one another when genetically removed as opposed to chemically inhibited^62^.

The robust inhibition of NB cell lines by ATR inhibition was confirmed in a xenograft NB model, where BAY 1895344 monotherapy treatment was as effective as the third-generation ALK TKI lorlatinib in reducing tumour growth (Fig. 5). However, while xenograft models provide in vivo information, they do not present the considerable therapeutic challenge that *Alk/MYCN*-driven genetically modified mice models provide. We reasoned that the effect of combining ALK and ATR inhibition in our GEMM models may be more marked than in our in vitro assays, leading us to devise a 14 day combination protocol incorporating both lorlatinib (targeting ALK) and BAY 1895344 (targeting ATR) (Fig. 6). Remarkably, use of BAY 1895344 in this 14 day (3 day on/4 day off) treatment protocol with lorlatinib led to the complete resolution of ALK-driven NB in two independent GEMMs (*Rosa26_Alkal2;Th-MYCN* and *Alk-F1178S;Th-MYCN*) with no detectable side effects (Fig. 6). Treatment with BAY 1895344 was accompanied by a strong apoptotic response as well as a robust immune response, with tumours heavily infiltrated with CD68-positive macrophages (Fig. 7). Moreover, 6/7 mice remain tumour free and healthy after this short 14-day treatment in the absence of any additional treatment. This can be compared with, e.g. the ALK TKI lorlatinib, which leads to a robust initial inhibition of rate of tumour growth in ALK-driven NB models^46,49^, but is unable to deliver a complete response as was observed here with BAY 1895344. The dramatic response we observed in both *Rosa26_Alkal2;Th-MYCN* and *Alk-F1178S;Th-MYCN* ALK-driven NB GEMMs is interesting in comparison to the results observed in the xenograft setting, where cessation of treatment resulted in tumour regrowth. One hypothesis is that the remarkable response in the *Rosa26_Alkal2;Th-MYCN* and *Alk-F1178S;Th-MYCN* ALK-driven NB GEMMs stems from their intact immune system. This is supported by the heavy infiltration of immune cells we observed in treated tumours (Fig. 7) and merits further investigation in the future. While BAY 1895344 has been explored both as monotherapy and in combination with PARP inhibitors, the NB patient population is paediatric and therefore great care must be taken to consider toxicity. Importantly, the recently reported phase I clinical trial of BAY 1895344 in adult solid tumours also employed a 3 day on/4 day off treatment regimen that was tolerated, supporting use in the clinic^35^. Altogether, our findings show that the recently described ATR inhibitor, BAY 1895344, potently inhibits growth of ALK-driven NB cells and xenografts. Moreover, aggressive *Alk/MYCN*-driven mouse NB tumour models respond completely and maintain a long-term tumour-free response. These data suggest that ATR may present an exploitable Achilles heel in NB, and strongly motivate continued exploration of ATR as a therapeutic target.

## Methods

### Antibodies and inhibitors

Primary antibodies against ATR (#13934, 1:1000), pATR (s428, #2853, 1:1000), ATM (#2873, 1:1000), pATM (#13050, 1:1000), CHK1(#2345, 1:1000), pCHK1(S345,#2348, 1:1000), pAKT (S473,#4060, 1:4000), pERK1/2 (Y204/T202, #4377, 1;2000), pALK (Y1604, #3341, 1:1000), β-Actin (#4970, 1:10,000), AKT (#9272), FOXM1 (#20459, 1:1000), pFOXM1 (T600, #14655, WB 1:100, IHC 1:1000), Ki67 (#12202, 1:800), α-tubulin (#2125, 1:10,000), cleaved caspase-3 (#9661, 1:200), p21 (#2947, 1:50), Survivin (#2808, 1:400), phosphor histone H3 (9701, 1:200), GAPDH (#5174, 1:20,000), p53 (#2527, 1:1000) and PARP (#9542, 1:1000) were obtained from Cell Signaling Technology. Phospho-ATM/ATR Substrate Motif [(pS/pT) QG] MultiMab™ Rabbit mAb mix (#6966) was also from Cell Signaling Technology. Primary antibodies detecting SUN2 (ab124916, 1:5000), CD68 (#ab125212; 1:100) and TOP2A (#ab52934, 1:1000) were purchased from Abcam and from Atlas Antibodies (HPA001209). Pan-ERK1/2 antibody (#610123, 1:10,000) was purchased from BD Transduction Laboratories (Franklin Lakes, NJ). Monoclonal antibody 135 (anti-ALK, 1:100) was produced in-house against the extracellular domain of ALK^66^. Horseradish peroxidase-conjugated secondary antibodies, goat anti-mouse immunoglobulin G (IgG) (# 32230), and goat anti-rabbit IgG (# 32260, 1:5000) were purchased from Thermo Fisher Scientific. Crizotinib (S1068), lorlatinib (S7536), etoposide (S1225), teniposide (S1787) and BAY 1895344 (S9864) were purchased from Selleck Chemicals.

### Cell culture

NB cell lines CLB-BAR, CLB-GE, CLB-GAR, SK-N-AS, IMR-32, NB-1, SHSY5Y, Kelly and SK-N-BE(2) were employed in this study. Cell lines are described in detail in Supplementary Table 1. CLB-BAR (gain of function, *Δexon4–11* truncated *ALK*), CLB-GE (gain of function, *ALK-F1174V* mutation) and CLB-GAR (gain of function, *ALK-R1275Q* mutation, 11q deletion) were obtained from The Centre Leon Berard, France under MTA. These cells were cultured on collagen coated plates. IMR-32 (wild-type ALK, ligand-dependent activation), and SK-N-AS (wild-type, non-amplified ALK) cell lines were purchased from ATCC. All above mentioned cell lines were cultured in complete media, RPMI 1640 supplemented with 10% fetal bovine serum (FBS) and a mixture of 1% penicillin/streptomycin at 37 °C and 5% CO_2_. For synchronisation, cells were seeded on pre-coated collagen plates at 37 °C and 5% CO_2_ overnight. Thymidine was added to the final concentration of 2 mM for 24 h. After 24 h, cells were washed with fresh media prior treatment with either DMSO or 50 mM BAY 1895344 inhibitor. For siRNA transfection, CLB-BAR cells were transfected with one of three duplex siRNAs targeting ATR (Stealth RNAi, Invitrogen) according to the manufacturer’s protocols. Cells transfected with scrambled siRNA (Invitrogen) were used as negative controls.

### Immunoprecipitation and immunoblotting

For immunoprecipitation, NB cells were seeded on collagen coated plates. The following day cells were treated with either lorlatinib or BAY 1895344 for 6 h. Cells were lysed with RIPA buffer and 1 mg per condition was were used for immunoprecipitated with 15 μl psmATR antibodies (Phospho-ATM/ATR Substrate Motif [(pS/pT) QG]) conjugated on beads. Remaining lysates were employed as loading controls. Immunoprecipitated samples were incubated overnight at 4 °C, subsequently washed (5 × 10 min at 4 °C) with RIPA buffer, then eluted and boiled in 50 µl SDS sample buffer. For immunoblotting, protein lysate and immunoprecipitation samples were separated on 7.5% bis-acryl-tris gels, transferred to polyvinylidene difluoride membranes (Millipore), blocked in 3% bovine serum albumin (BSA) (phosphoprotein blots) and immunoblotted with primary antibodies as indicated overnight at 4 °C. Secondary antibodies were diluted 1:10,000 and incubated at room temperature for 1 h. Enhanced chemiluminescence substrates were used for detection (GE Healthcare), and membranes were scanned using LI-COR Odyssey instrumentation. For psmATR immunoprecipitations, the Image Studio Lite version 5.2 software was used to quantify immunoblot bands and statistical analysis was performed in GraphPad Prism9. Experiments were performed as four replicates.

### IC_50_ determination

CLB-BAR and CLB-GE cells were seeded on 48-well plates. Next day, cells were treated with increasing concentration of BAY 1895344 inhibitor (25, 50, 100, 150, 200, 300, 500 and 1000 nM) and analysed in an IncuCyte^®^ Live Cell Analysis system (Essen BioScience) for 3 days, where images were taken every 24 h. This experiment was performed in three independent biological replicates.

### Apoptosis assay

NB cells were seeded and treated after 24 h with BAY 1895344 (50 and 100 nM) and crizotinib (250 nM) as a positive control for 24 h. Cells were lysed with RIPA buffer and protein concentration was determined by BCA assay. Samples were run and immunoblotted with PARP antibody, which recognises both full length and cleaved PARP, cleaved caspase 3 and p53. Tubulin or GAPDH were used to normalise cleaved PARP.

### RNA-Seq analysis

#### Cell line RNA-Seq

CLB-BAR and CLB-GE cells were seeded on 15 cm^3^ dishes in triplicate prior to treatment with 50 nM BAY 1895344 for 24 and 48 h. Control samples were harvested after 48 h. RNA was extracted and RNA-Seq performed by Novogene UK.

#### Mouse tumour RNA-Seq

*Rosa26_Alkal2Tg/0;TH-MYCNTg/0* and *Alk-F1178SKI/0;TH-MYCNTg/0* on a *129×1/SvJ* background (JAX stock #000691) (*n* = 4 per genotype) were screened by ultrasound, 2–3 times a week from approximately 35 days of age. Tumours were followed until they reached a size of 8–12 mm in average diameter after which a 3D scan of the tumour was made and treatment started with BAY 1895344 25 mg/kg b.i.d. P.O. for 3 days. A 3D scan was repeated at day 3, the mice were sacrificed and tumour extracted and incubated in RNAlater (Thermo Fisher #AM7020). RNA was extracted using the ReliaPrep™ RNA Miniprep Systems (Promega #6111) according to the manufacturer’s protocol. The purified RNA was sent to Novogene UK for library preparation and sequencing. Images and 3D measurements were analysed in VevoLab (Fujifilm VisualSonics, Toronto, Canada).

RNA-Seq paired-end reads (read length 150 base pairs) were aligned to the GRCh38 (human cell line data) or GRCm38 (mice data) reference genome using hisat2^67^. The average alignment efficiency was 90.9% and 90.3% for human and mice data, respectively. Genes were annotated using GENCODE 29 (human) or M22 (mouse)^68^ and quantified using HTSeq^69^. Only coding genes were used for further analysis. Differential gene expression was determined using DESeq2^70^. Genes were considered differentially expressed if their absolute log2 fold change values were above 1.5 at FDR-adjusted *p* values (*Padj*) below 0.01, considering a hyperbolic threshold given by:

1 $${\log }_{10}P{{adj}}={\log }_{10}P{{ad{j}}}_{{{{{{\mathrm{TH}}}}}}}+\frac{c}{\sqrt{{({\log }_{2}{{FC}})}^{2}-{({\log }_{2}{{F{C}}}_{{{TH}}})}^{2}}}$$

With Padj_TH_ and log2FC_TH_ representing the adjusted *p* value (0.01) and log2 fold change (1.5) thresholds, respectively, and *c* the curve parameter (here *c* = 0.5).

### Preparation of samples for proteomics and phosphoproteomics analyses

For total proteomics, 10.5 × 10^6^ NB cells (CLB-BAR or CLB-GEMO) were seeded in FBS-free RPMI 1640 cell culture media in 15 cm^3^ dishes. One 15 cm^3^ dish of cells was used for each replicate, and experiments were performed in triplicate. After 24 h, cells were treated either with BAY 1895344 (50 nM) for 24 or 48 h or with DMSO control for 48 h. Cells were harvested by washing with PBS, briefly trypsinising and then neutralising with non-FBS containing media. Aliquots were removed to perform immunoblotting validation and the remaining cells isolated by centrifugation at 200*g*, for 6 min. Pellets were resuspended in 1 ml of ice-cold PBS, and cell washing was repeated 5 times. Cell pellets were stored at −80 °C for subsequent analysis.

Phosphoproteomics was performed on cells synchronised by thymidine block (2 mM thymidine, 24 h). Briefly, 10.5 × 10^6^ NB cells (CLB-BAR or CLB-GEMO) were seeded in FBS-free RPMI 1640 cell culture media in 15 cm^3^ dishes. One 15 cm^3^ dish of cells was used for each replicate, and experiments were performed in triplicate. After 24 h, cells were either (i) non-treated or (ii) subjected to thymidine block (2 mM thymidine, 24 h) or (iii) subjected to thymidine block (2 mM thymidine, 24 h), then washed 2x with FBS-free RPMI and released for 6 h or (iv) subjected to thymidine block (2 mM thymidine, 24 h), then washed 2x with FBS-free RPMI, then released for 6 h in the presence of BAY 1895344 (50 nM). Cells were harvested by washing with PBS, briefly trypsinising and then neutralising with non-FBS containing media. Aliquots were removed to perform immunoblotting validation and the remaining cells isolated by centrifugation at 200*g*, for 6 min. Pellets were resuspended in 1 ml of ice-cold PBS, and cell washing was repeated 5 times. Cell pellets were stored at −80 °C for subsequent analysis.

### Sample preparation for proteomic analysis

Samples were homogenised using lysis matrix D (1.4 mm ceramic spheres) on a FastPrep^®^-24 instrument (MP Biomedicals, OH, USA) for five repeated 40 s cycles at 6.5 m/s in 300 µl of the buffer containing 2% sodium dodecyl sulfate (SDS), 50 mM triethylammonium bicarbonate (TEAB) and 1X Pierce Phosphatase inhibitor (A32957, Thermo Fischer Scientific, Waltham, MA, USA). Lysed samples were centrifuged at 21,100*g* for 10 min and the supernatants were transferred to clean tubes. The lysis tubes were washed with 200 µl of the lysis buffer, centrifuged at 21,100*g* for 10 min, the supernatants were combined with the corresponding lysates from the previous step. Protein concentrations in the lysates were determined using Pierce™ BCA Protein Assay Kit (Thermo Fischer Scientific) and the Benchmark™ Plus microplate reader (Bio-Rad Laboratories, Hercules, CA, USA) with bovine serum albumin (BSA) solutions as standards.

Aliquots containing 200 µg of total protein from each sample were incubated at 37 °C for 60 min in the lysis buffer with DL-dithiothreitol (DTT) at 100 mM final concentration. The reduced samples were processed using the modified filter-aided sample preparation (FASP) method^71^. In short, the reduced samples were diluted to 1:4 by 8 M urea solution, transferred onto Nanosep 30k Omega filters (Pall Corporation, Port Washington, NY, USA) and washed 4 times by adding 200 µl of 8 M urea and subsequent centrifugation at 13,800*g*. Free cysteine residues were modified using 10 mM methyl methanethiosulfonate (MMTS) solution in digestion buffer (0.5% sodium deoxycholate (SDC), 50 mM TEAB) for 30 min at room temperature and the filters were then repeatedly washed with 100 µl of digestion buffer. Pierce trypsin protease (MS Grade, Thermo Fisher Scientific) in digestion buffer was added at a ratio of 1:100 relative to total protein mass and the samples were incubated at 37 °C for 3 h; another portion of trypsin (1:100) was added and the mixture was incubated at 37 °C overnight. The peptides were collected by centrifugation and labelled using Tandem Mass Tag (TMTpro 10 plex and 16plex) reagents (Thermo Fischer Scientific) according to the manufacturer’s instructions. The labelled samples were combined into one pooled sample, concentrated using vacuum centrifugation, and SDC was removed by acidification with 10% TFA and subsequent centrifugation. The labelled pooled sample was treated with Pierce peptide desalting spin columns (Thermo Fischer Scientific) according to the manufacturer’s instructions.

Out of 2.80 mg of peptide material in the pooled labelled sample, an aliquot corresponding to 400 µg was withdrawn for the total proteome analysis, an aliquot of 1.20 mg was subjected to phosphopeptide enrichment using the High-Select Fe-NTA Enrichment Kit and another 1.20 mg aliquot was treated with the High-Select TiO_2_ Phosphopeptide Enrichment Kit (both Thermo Fisher Scientific) according to the manufacturer’s instruction. The eluted phosphopeptide samples were pooled.

For the total proteome analysis, the corresponding aliquot was fractionated into 40 primary fractions by basic reversed-phase chromatography (bRP-LC) using a Dionex Ultimate 3000 UPLC system (Thermo Fischer Scientific). Peptide separations were performed on a reversed-phase XBridge BEH C18 column (3.5 μm, 3.0 × 150 mm, Waters Corporation) using a linear gradient from 3% to 40% solvent B over 18 min followed by an increase to 100% B over 5 min and hold at 100% B for min. Solvent A was 10 mM ammonium formate buffer at pH 10.00 and solvent B was 90% acetonitrile, 10% 10 mM ammonium formate at pH 10.00. The primary fractions were concatenated into final 20 fractions (1+21, 2+22, … 20+40), evaporated and reconstituted in 20 μl of 3% acetonitrile, 0.2% formic acid for nanoflow LC-MS analysis. The enriched phosphopeptide sample was fractionated into 20 primary fractions on the same LC setup with the gradient from 3% to 40% solvent B over 12 min, from 40% to 100% B over 4 min and 100% B for 6 min, the primary fractions were concatenated into final 20 fractions (1+11, 2+12, … 10+20), evaporated and reconstituted in 15 μl of 3% acetonitrile, 0.2% formic acid for nanoflow LC-MS analysis.

### LC-MS/MS analysis

The fractions were analysed on an Orbitrap Fusion Lumos Tribrid mass spectrometer interfaced with an Easy-nLC 1200 liquid chromatography system (both Thermo Fisher Scientific). Peptides were trapped on an Acclaim Pepmap 100 C18 trap column (100 μm × 2 cm, particle size 5 μm, Thermo Fischer Scientific) and separated on an analytical column (75 μm × 35 cm, packed in-house with Reprosil-Pur C18, particle size 3 μm, Dr. Maisch, Ammerbuch, Germany) at a flow of 300 nl/min using 0.2% formic acid in water as solvent A and 80% acetonitrile, 0.2% formic acid as solvent B.

For the total proteome analysis, peptides were eluted using a linear gradient from 5% to 33% B over 77 min followed by an increase to 100% B over 3 min and a hold at 100% B for 10 min. MS scans were performed at 120,000 resolution in the *m*/*z* range 375–1500. The most abundant doubly or multiply charged precursors from the MS1 scans were isolated using the quadrupole with 0.7 *m*/*z* isolation window with a “top speed” duty cycle of 3 s and dynamic exclusion within 10 ppm for 45 s. The isolated precursors were fragmented by collision-induced dissociation (CID) at 30% collision energy with the maximum injection time of 60 ms and detected in the ion trap, followed by multinotch (simultaneous) isolation of the top 10 MS2 fragment ions within the *m*/*z* range 400–1400, fragmentation (MS3) by higher-energy collision dissociation (HCD) at 55% collision energy and detection in the Orbitrap at 50,000 resolution, *m*/*z* range 100–500 and maximum injection time 120 ms.

For the phosphorylation analysis, peptides were eluted using a linear gradient from 5% to 35% B over 75 min followed by an increase to 100% B over 5 min and a hold at 100% B for 10 min. MS scans were performed at 120,000 resolution in the *m*/*z* range 375–1375. The most abundant doubly or multiply charged precursors from the MS1 scans were isolated using the quadrupole with 0.7 *m*/*z* isolation window with a “top speed” duty cycle of 3 s and dynamic exclusion within 10 ppm for 60 s. The isolated precursors were fragmented by HCD at 38% collision energy and the MS2 spectra were detected in the Orbitrap at 50,000 resolution, with the fixed first *m*/*z* 100 and maximum injection time 105 ms.

### Proteomic data analysis

Identification and relative quantification was performed using Proteome Discoverer version 2.4 (Thermo Fisher Scientific; Supplementary Dataset 3). The database search was performed using the Mascot search engine v. 2.5.1 (Matrix Science, London, UK) against the Swiss-Prot Homo sapiens database. Trypsin was used as a cleavage rule with no missed cleavages allowed; methylthiolation on cysteine residues, TMTpro at peptide N-termini and on lysine side chains were set as static modifications, and oxidation on methionine was set as a dynamic modification. For the total proteome analysis, precursor mass tolerance was set at 5 ppm and fragment ion tolerance at 0.6 Da. For the phosphopeptide analysis, precursor mass tolerance was set at 5 ppm and fragment ion tolerance at 30 mmu; phosphorylation on serine, threonine, and tyrosine, and deamidation and acetylation were set as additional dynamic modifications. Data matching was performed with up to 2 missed tryptic cleavages. Percolator was used for PSM validation with the strict FDR threshold of 1% for phosphoproteomics and 5% for proteomics.

Quantification was performed in Proteome Discoverer 2.4. TMT reporter ions were identified with 3 mmu mass tolerance in the MS2 HCD spectra for the phosphopeptide experiment or in the MS3 HCD spectra for the total proteome experiment, and the TMT reporter S/N values for each sample were normalised within Proteome Discoverer 2.4 on the total peptide amount. Only the unique identified peptides were taken into account for the protein quantification.

Differential protein expression was determined using the R Differential Enhancement of Proteomics data (DEP) package (default settings)^72^. Proteins/phosphosites were considered differentially expressed/phosphorylated when the absolute log2 fold change values were above 0.3 at FDR-adjusted *p* values below 0.05, considering a hyperbolic threshold as described higher.

Amino acid sequences ±5 amino acids adjacent to phosphorylation sites were extracted from Uniprot using the R *protr* package^73^. S/T Q sites were defined as S or T phosphorylation sites followed by a downstream (position +1). Sequence logo plots were generated using the kpLogo method available at http://kplogo.wi.mit.edu^74^. Position-specific amino acid enrichments were determined for sequences extending from 5 amino acids upstream to 5 amino acids downstream of the hypo- and hyperphosphorylated sites, respectively. Sites without differential phosphorylation signals were used as a background sequence.

### Gene set enrichment analysis

Preranked gene set enrichment analyses (GSEA) were performed using the R *fgsea* package (*fgseaMultilevel* function, default parameters) with ranking based on the DEseq2 statistic (RNA-Seq) or log2FC value (proteomics). Unranked GSEA was performed using Fisher’s exact test followed by FDR correction using the Benjamini-Hochberg method^75^. Hallmark and Canonical pathway gene sets were downloaded from the Molecular Signatures Database v7.2^76^ and transcription factor target information was derived from RegNetwork^77^, downloaded from www.regnetworkweb.org.

### STRING protein–protein interaction network analysis

Human protein–protein interaction data were download from STRING (v11, https://stringdb-tatic.org/download/protein.links.v11.0/9606.protein.links.v11.0.txt.gz)^78^. Only high-confidence interactions (combined score > 0.7) were kept for downstream analysis. All directly interacting proteins with minimally one hypophosphorylated site were plotted using the R *igraph* package.

### Xenograft neuroblastoma model

Female *BALB/cAnNRj-Foxn1nu* mice (Janvier Laboratory), 4–6 weeks old, were housed with access to food and water ad libitum in a 12:12 light–dark cycle. Animals were allowed to acclimatise for 1 week prior to being subcutaneously injected into the left flank with 1 × 10^6^ CLB-BAR cells in serum-free medium mixed with Matrigel Matrix at a ratio of 1:1. The total injection volume was 100 µl. Once the tumour reached a volume of 150 mm^3^, mice were randomised to treatment groups. Compounds were administered orally at 10 mg/kg bodyweight twice daily for lorlatinib (20 mg/kg per day) and 50 mg/kg bodyweight for BAY1895344 twice daily (100 mg/kg per day) for 14 days, according to the following schedule: 3 day on/4 day off. The control group was treated with vehicle solution; a mix of 2% DMSO and 30% PEG300. Tumour volume was measured by caliper every 2 days and calculated by the following equation: V = (π/6) × L × W^2^ (V, volume; L, length; W, width). TGI was calculated according to the following formula:

2 $${{If}}\,{{T{V}}}_{{{{{{\rm{t}}}}}}} > {{T{V}}}_{0},\,{{TGI}}=100 \% \times \left(1-\frac{{{T{V}}}_{{{{{{\rm{t}}}}}}}-{{T{V}}}_{0}}{{{C{V}}}_{{{{{{\rm{t}}}}}}}-{{C{V}}}_{0}}\right)$$

3 $${{If}}\,{{T{V}}}_{{{{{{\rm{t}}}}}}} < {{T{V}}}_{0},\,{{TGI}}=100 \% \times \left(2-\frac{{{T{V}}}_{{{{{{\rm{t}}}}}}}}{{{T{V}}}_{0}}\right)$$

where TV_0_ was the tumour volume in the treatment group at the beginning of the study, TV_t_ was the tumour volume in the treatment group at the end of the study, CV_0_ was the tumour volume in the control group at the beginning of the study, and CV_t_ was the tumour volume in the control group at the end of the study. Animal weight was recorded every 2 days. Humane endpoints were a tumour size exceeding 20 mm or if the animal showed symptoms due to tumour burden, mice that met these criteria were sacrificed. All experimental procedures and protocols were performed in accordance with the Regional Animal Ethics Committee approval, Jordbruksverket (1890–2018, 3225–2020).

### Transgenic mouse model survival

*Rosa26_Alkal2Tg/0;TH-MYCNTg/0* (*n* = 15, mixed sex) and *Alk-F1178SKI/0;TH-MYCNTg/0* (*n* = 7, mixed sex) on a *129×1/SvJ* background (JAX stock #000691) were screened by ultrasound, 2–3 times a week from approximately age 35 days. Tumours were followed until a size of 3–6 mm in average diameter, at which a 3D scan of the tumour was made and treatment started (Day 0). Screening and 3D image acquisition a Vevo 3100 imaging systems (FUJIFILM VisualSonics, Toronto, Canada). The VisualSonics MX550D (25–55 MHz, 40 μm axial resolution) linear array transducer was used for all image acquisition. For 3D scanning, the probe was attached to a step motor, animals were anaesthetised with isoflurane and respiration rate, ECG and body temperature were monitored during the procedure. Images were analysed in VevoLab (Fujifilm VisualSonics, Toronto, Canada). Mice were treated P.O. twice daily with 10 mg/kg lorlatinib for 30 days or a combination regimen (*n* = 4 for each experimental arm). Combination treatment consisted of 3 days BAY 1895344 25 mg/kg P.O. b.i.d., 4 days lorlatinib 10 mg/kg b.i.d., 3 days of both inhibitors as previously and 4 days of monotreatment with lorlatinib, for a total of 14 days. At the end of treatment mice were followed until tumour could be clearly observed without palpitation, deteriorated health of the mice or sudden death. Humane endpoints were a tumour size exceeding 20 mm or if the animal showed symptoms due to tumour burden, mice that met these criteria were sacrificed. All experimental procedures and protocols were performed in accordance with the Regional Animal Ethics Committee approval, Jordbruksverket (1890–2018, 3225-2020).

### Histological analysis of tumour sections

Tumour tissue were fixed in 4% (vol/vol) phosphate buffered formaldehyde (Histolab Products, Gothenburg, Sweden), embedded in paraffin, and sections of 5 μm were prepared by using microtome. Paraffin sections were stained with hematoxylin and eosin (H&E; Histolab Products) for morphological analysis. Proliferation, ATR downstream signalling components, apoptotic induction, G2/M checkpoint and immune cell markers (pan-macrophage) were analysed by immunohistochemistry of Ki67, pFOXM1, CC3, CD68, p21, TOP2A, Survivin, and phosphohistone H3, primary antibodies were detected with SignalStain^®^ Boost IHC Detection Reagent (HRP, Rabbit) (#8114, Cell Signaling Technology), developed with SignalStain^®^ DAB Substrate Kit (#8059, Cell Signaling Technology) and Mayers counterstained with hematoxylin (#01820, Histolab Products AB).

### Statistical analysis

Statistical analyses were performed with either GraphPad Prism 7/8 software or R statistical package (v4.0). Statistical tests are indicated in the respective sections and figure captions. Multiple testing corrections were performed using the Benjamini-Hochberg method^75^.

### Reporting summary

Further information on research design is available in the Nature Research Reporting Summary linked to this article.

## Supplementary information

Supplementary information file.

Description of Additional Supplementary files.

Supplementary Dataset 1.

Supplementary Dataset 2.

Supplementary Dataset 3.

Reporting summary.

## Data Availability

The mass spectrometry proteomics data are available in the ProteomeXchange Consortium via the PRIDE partner^79^ repository (dataset identifier PXD027187). RNA-Seq data are available in ArrayExpress (https://www.ebi.ac.uk/arrayexpress/, accession numbers E-MTAB-10603 & E-MTAB-10616). All other data required to evaluate the conclusions in the paper are provided in Supplementary information files. Source data are provided with this paper.

## Source data

Source Data.

## Code availability

Source code used for RNA-Seq, proteomics and phosphoproteomics analyses is available at GitHub https://github.com/CCGGlab/ATR and 10.5281/zenodo.5594083.
