# Supplementary material for: ATR inhibition enables complete tumour regression in ALK-driven NB mouse models
Source: Nat Commun. 2021 Nov 24;12:6813. doi: 10.1038/s41467-021-27057-2 (PMC8613282; doi:10.1038/s41467-021-27057-2)

Supplementary Information for:

ATR inhibition enables complete tumour regression in ALK-driven NB mouse models

Szydzik *et al.*

Supplementary Table 1

Cell line	Genomic profile	*ALK* status (amp, mut ex 25-25)	*MYCN* status	*TP53* status (mut ex 5-9)
CLB-BAR	MNA	Amp. whole *ALK* exc. Part of i3 (non-amp). Note, exome sequencing shows del ex4-11	*MYCN* amplified	No mut in *TP53*
CLB-GE	MNA	*ALK* amp<br>Mutation F1174V	*MYCN* amplified	No mut in *TP53*
CLB-GAR	11q-del	2p-gain with break within *ALK* intron 1<br>Mutation R1275Q	Non *MYCN* amplified	No mut in *TP53*
Kelly	MNA + 11q-del	No amplification<br>2p-gain<br>Mutation F1174L	*MYCN* amplified	HomZ. mut P177T
NB-1	MNA	*ALK* amp	*MYCN* amplified	No mut in *TP53*
SHSY5Y	17q-gain	Mutation F1174L	Non *MYCN* amplified	No mut in *TP53*
IMR32	MNA	Ampl of *ALK* ex 3-4 only.<br>No mutation.	*MYCN* amplified	No mut in *TP53*
SK-N-AS	11q-del	No mutation<br>No amplification	Non *MYCN* amplified	No mut in *TP53*
SK-N-BE(2)	MNA	2p-gain<br>No mutation<br>No amplification	*MYCN* amplified	HomZ. mut C145F

Genomic profile data for the NB cell lines employed in this study.

Overview of genetic aberrations selected indicated genes and chromosomes for the nine NB cell lines used in this study. MNA, *MYCN*-amplified; HomZ., homozygote; mut, mutant; amp, amplification; ex, exon; del, deletion ; expr., expression[1].

1. Umapathy, G. *et al.* MEK inhibitor trametinib does not prevent the growth of anaplastic lymphoma kinase (ALK)-addicted neuroblastomas. *Science signaling* **10** (2017).

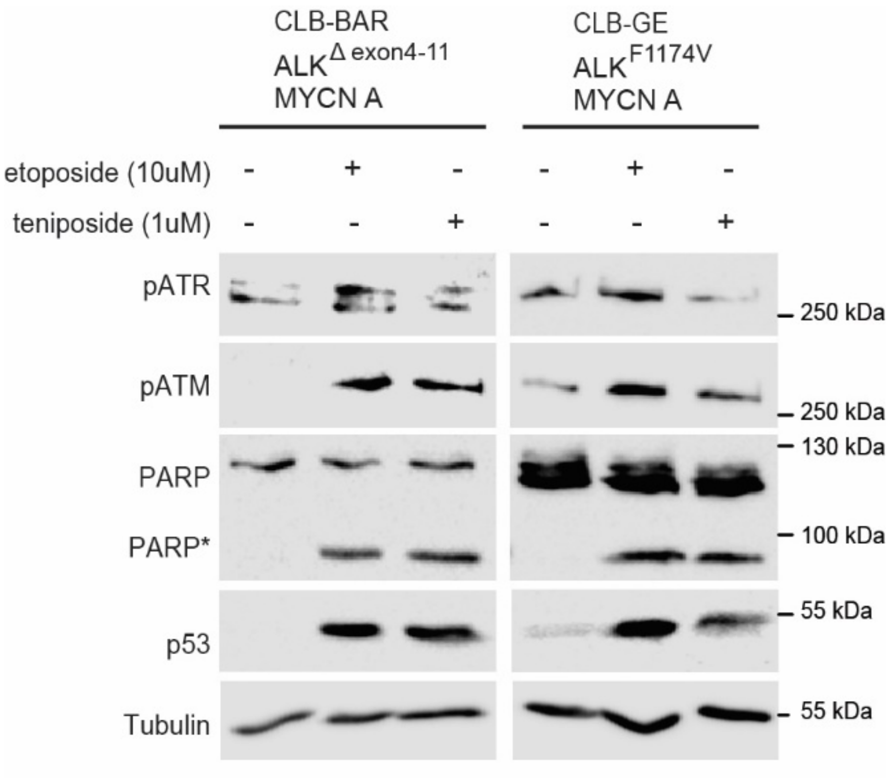

Supplementary Fig. 1 Treatment of NB cells with etoposide or teniposide. ALK positive NB cell lines CLB-BAR and CLB-GE were treated with etoposide or teniposide, as indicated. Cell lysates were immunoblotted for pATR, pATM, PARP/PARP* (cleaved PARP) and p53. Tubulin was employed as a loading control. n=3 biologically independent experiments.

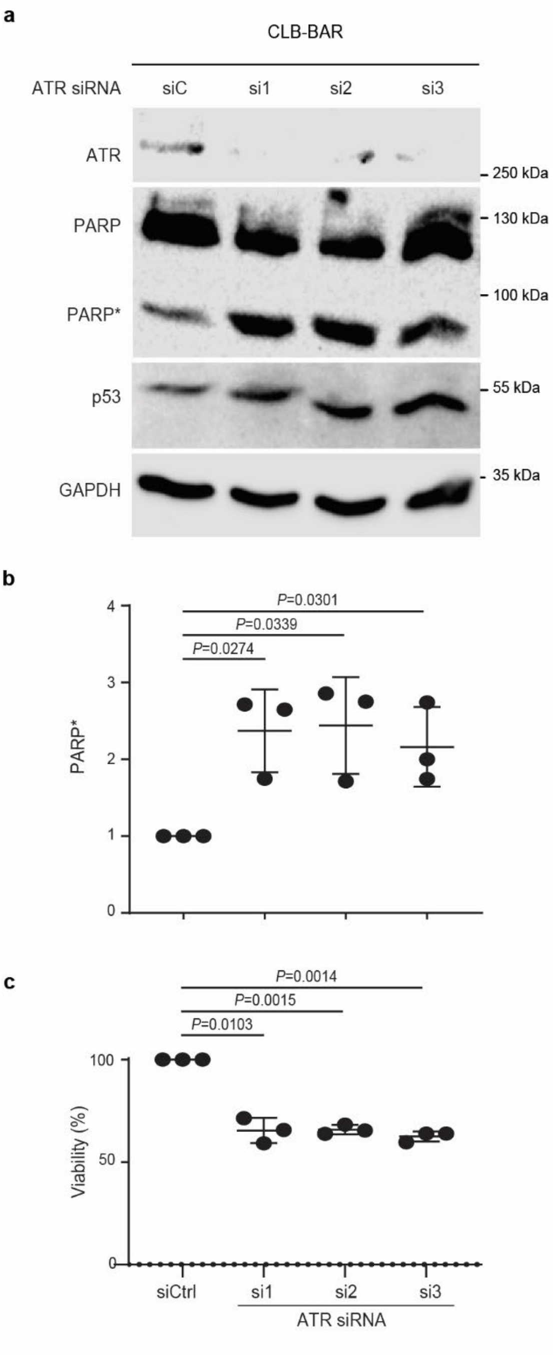

Supplementary Fig. 2 ATR siRNA treatment of NB cells. a CLB-BAR cells were transfected with either scrambled control siRNA (siC) or with one of three siRNAs against ATR (si1 to si3). Cell lysates were immunoblotted for ATR, PARP/cleaved PARP (PARP*) and p53. GAPDH was employed as loading control. Quantification of PARP* is shown in **b.** Data are presented as mean values ± SD. n=3 biologically independent experiments. Students paired t test, *P* values indicated. **c** CLB-BAR cells were transfected with double-stranded 25 nucleotide RNA duplexes targeting ATR. Viability of transfected cells was analyzed after 4 days using resazurin assay. Results are presented as percent viability of siRNA-transfected cells relative to scramble control. Data are presented as mean values ± SD. n=3 biologically independent experiments with each transfection performed in triplicate. Students paired t test, *P* values indicated.

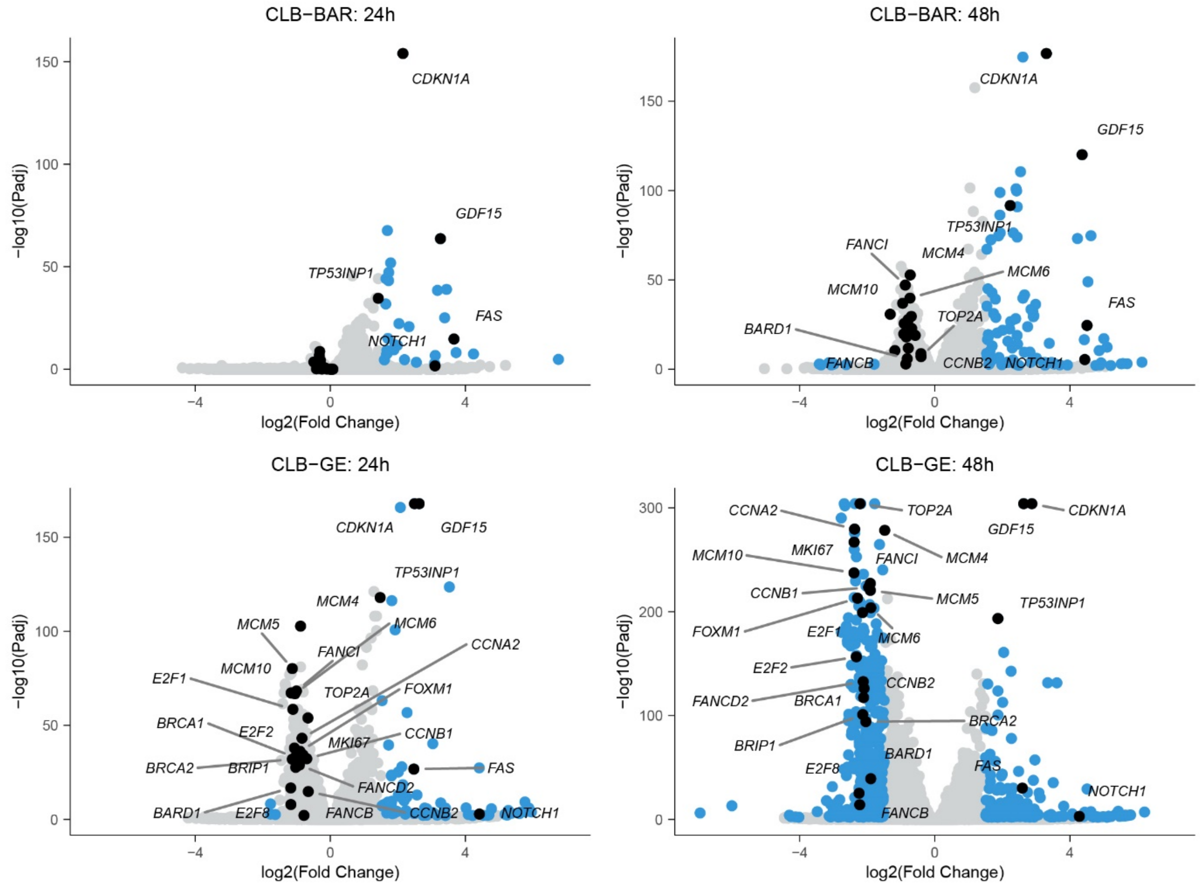

Supplementary Fig. 3 Transcriptomic response to BAY 1895344 treatment of NB cells. CLB-BAR and CLB-GE NB cells were treated for 24 or 48h with 50 nM BAY 1895344. Differential gene expression with untreated control conditions was determined using RNA-Seq. See Supplementary Dataset 1 for detailed results. Differentially expressed genes (log2FoldChange +/-1.5 at 1% FDR) indicated in blue on volcano plots, with the same genes as in main Fig. 3B indicated and labelled in black.

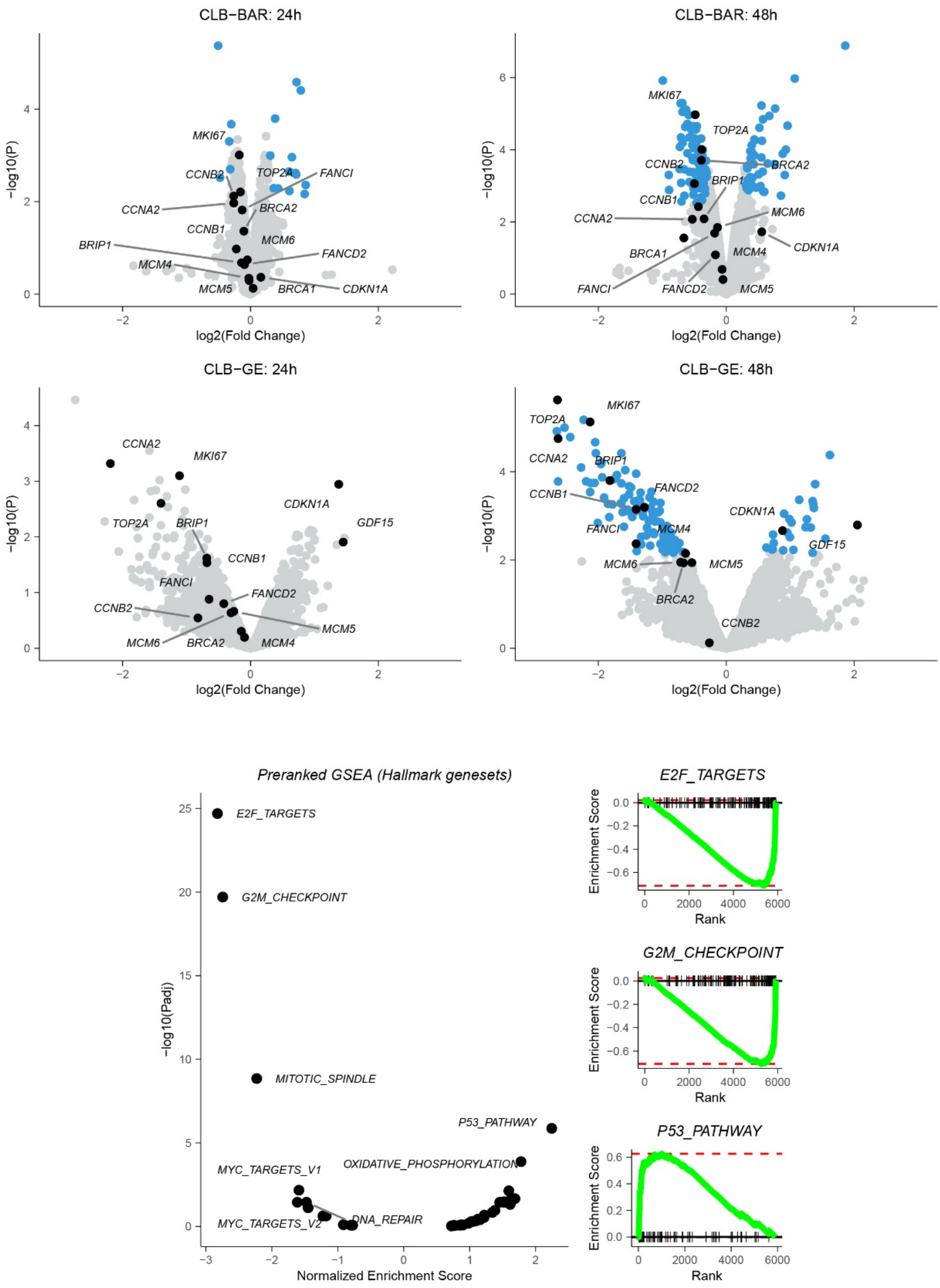

Supplementary Fig. 4 Proteomic response to BAY 1895344 treatment of NB cells. CLB-BAR and CLB-GE NB cells were treated for 24 or 48h with 50 nM BAY 1895344 and differential protein expression with untreated control conditions was determined using proteomics. See Supplementary Dataset 1 for detailed results. Differentially expressed proteins (log2 FoldChange +/-0.3 at 5% FDR) indicated in blue on volcano plots, with proteins corresponding to genes indicated in main Fig. 3B indicated and labelled in black. Lower plots represent preranked Hallmark GSEA results (CLB-GE response 48h). Lower left panel shows normalized enrichment scores and corresponding adjusted *P* values with labelling of enriched gene sets at 5% FDR. Right panels represent running score plots for the indicated gene sets.

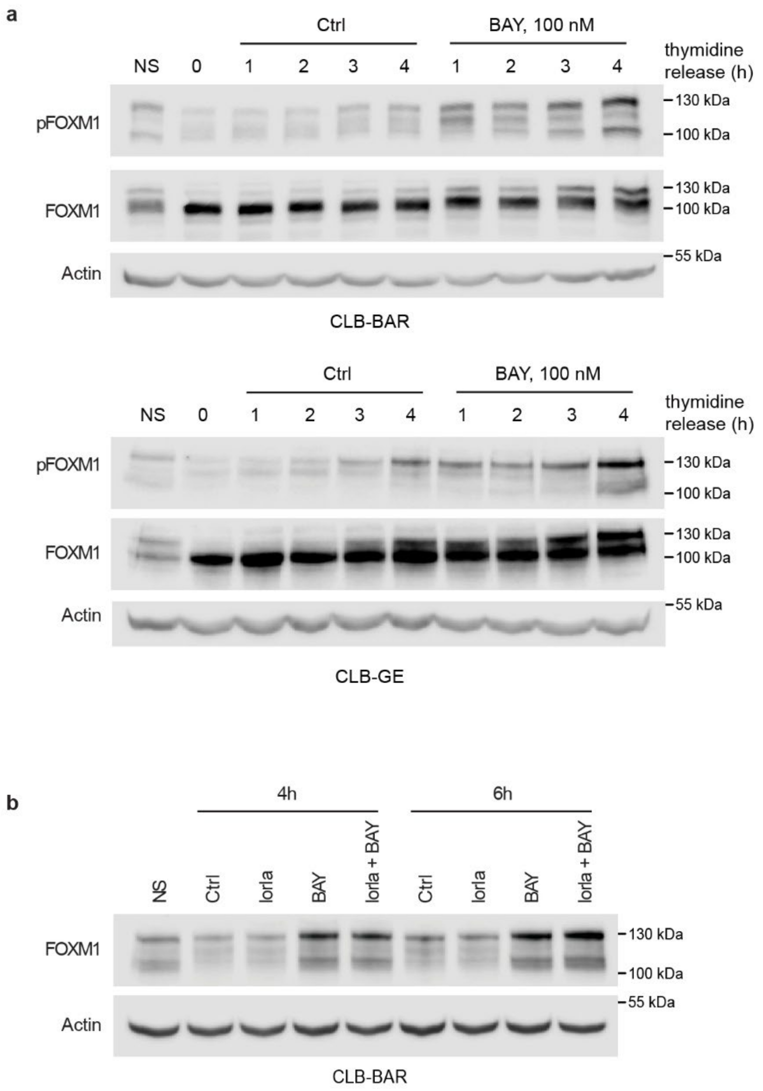

Supplementary Fig. 5 ATR and FOXM1 regulate the S/G2 checkpoint in NB cells. **a** CLB-GE and CLB-GE cells were synchronized in G1/S phase with thymidine prior to treatment with 100 nM BAY 1895344 for 0-4 h, as indicated. Cell lysates were immunoblotted for pFOXM1, FOXM1 and actin as loading control. **b** Synchronised CLB-BAR cells were released from thymidine block and treated with DMSO (Ctrl), BAY 1895344 (50 nM), lorlatinib (30 nM) and combination of BAY 1895344 and lorlatinib (50 nM and 30 nM, respectively) for 4 and 6 h. Lysates were immunoblotted for FOXM1 and actin as loading control. n=3 independent biological experiments.

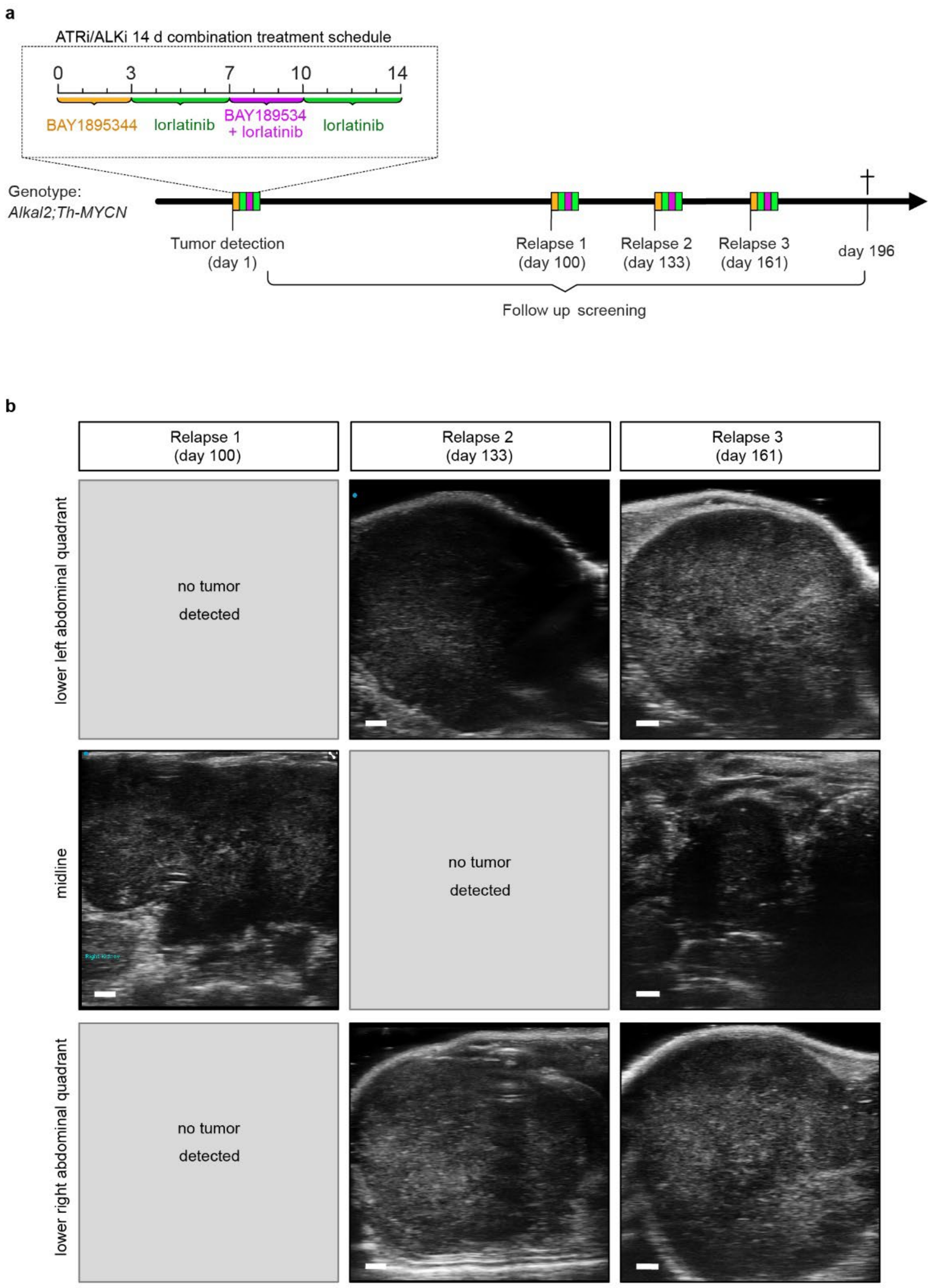

Supplementary Fig. 6 Detailed timeline of tumour incidence in the single relapsing BAY 1895344/lorlatinib treated mouse. a Timeline of tumour incidence, treatments, relapse and death of the single relapsing mouse (genotype: *Rosa26_Alkal2;Th-MYCN*). **b** Ultrasound images of tumours found at relapse (scale bars, approximately 1mm).

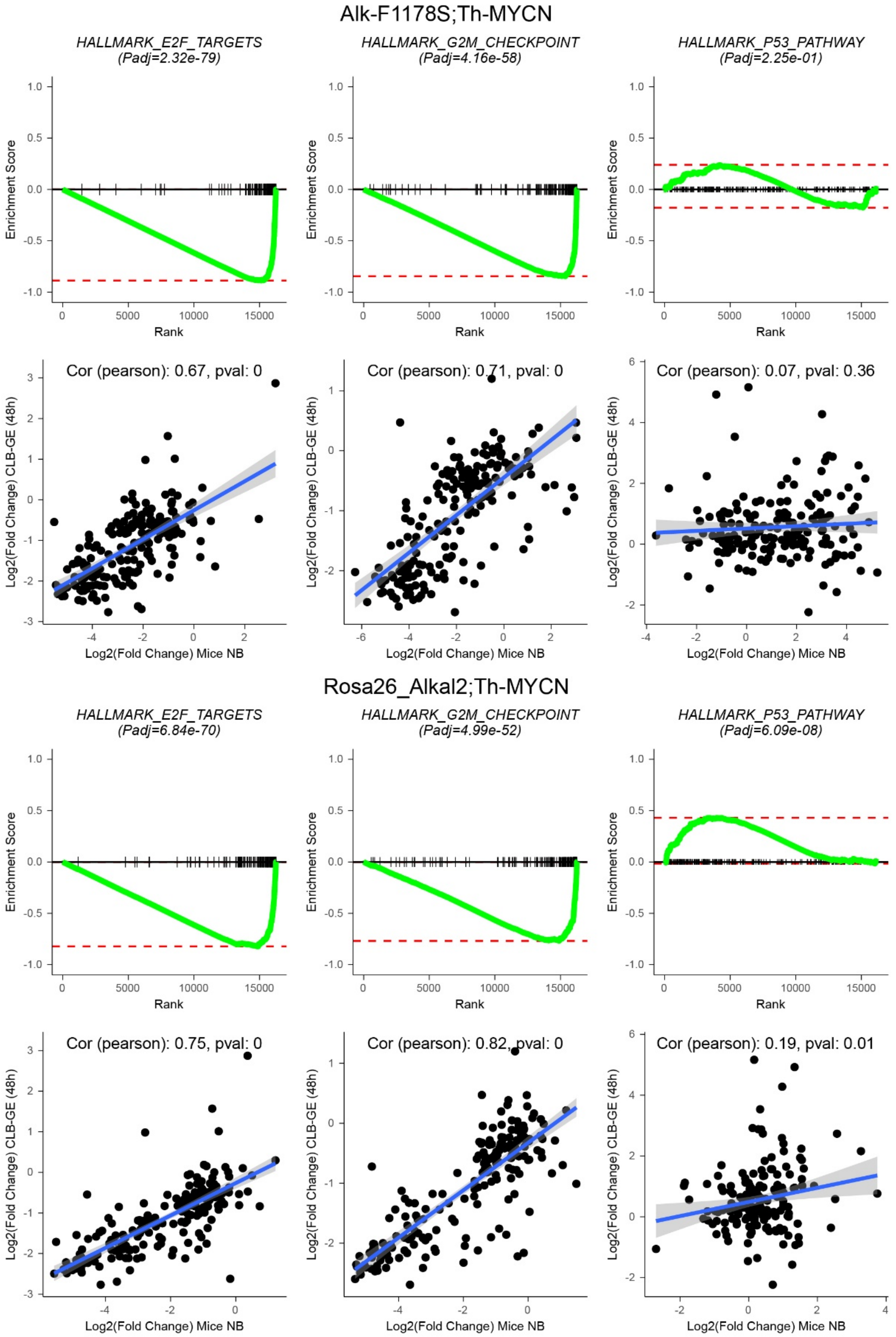

Supplementary Fig. 7 Correlation between transcriptomic responses to ATR inhibition in NB cell lines and genetic mouse models. Running score plots for 3 Hallmark gene sets and 2 genetic mouse models as indicated. Correlation plots between differentially expressed log2FoldChange values in mice tumours (x-axis) and CLB-GE NB cells (48h post-treatment, y-axis) for the same gene sets as above. Linear regression lines in blue with 95% confidence intervals in gray. Pearson correlation coefficients with corresponding student t-test P values indicated on top.

Source Data for Supplementary Fig. 1

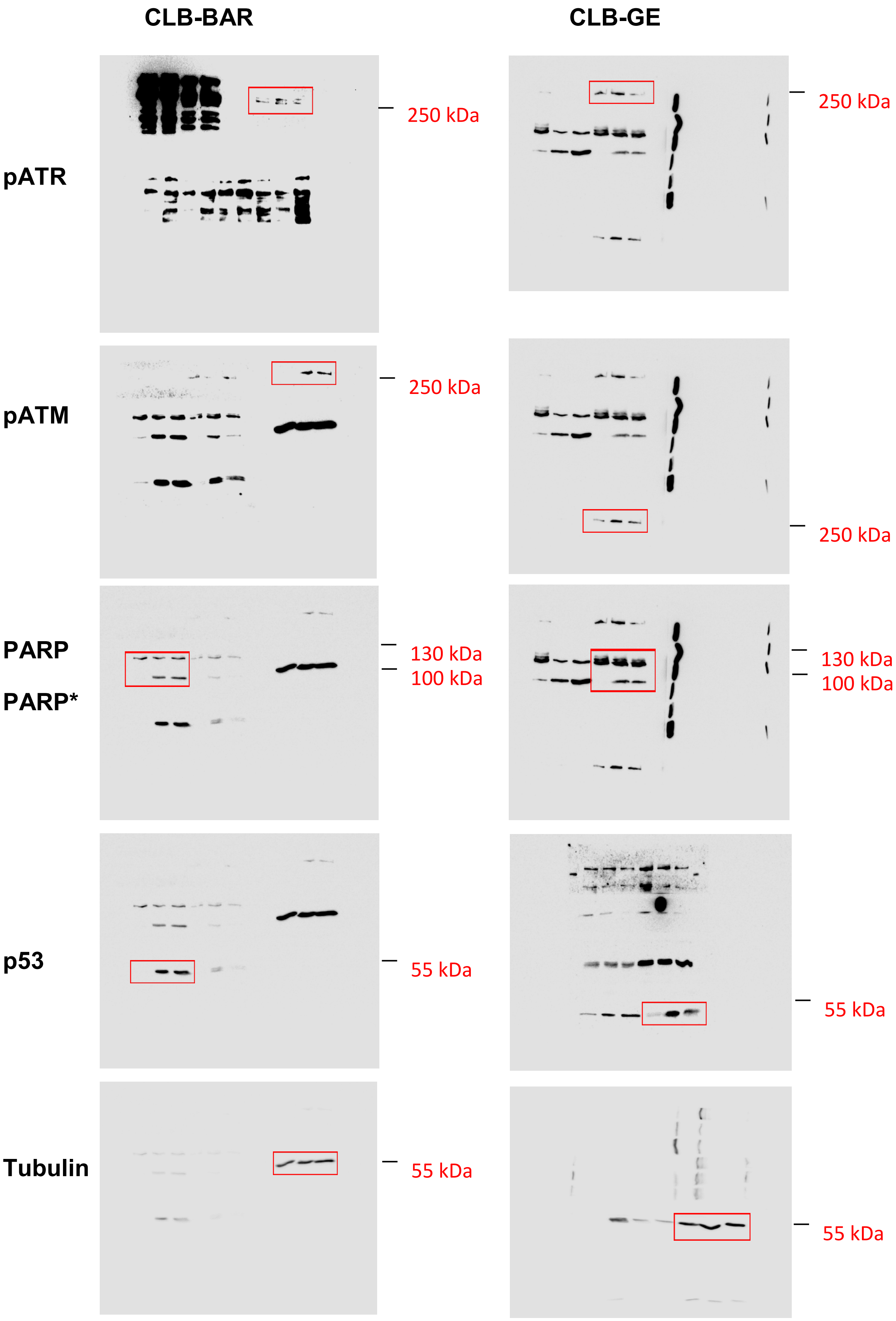

Source Data for Supplementary Fig. 2a

ATR

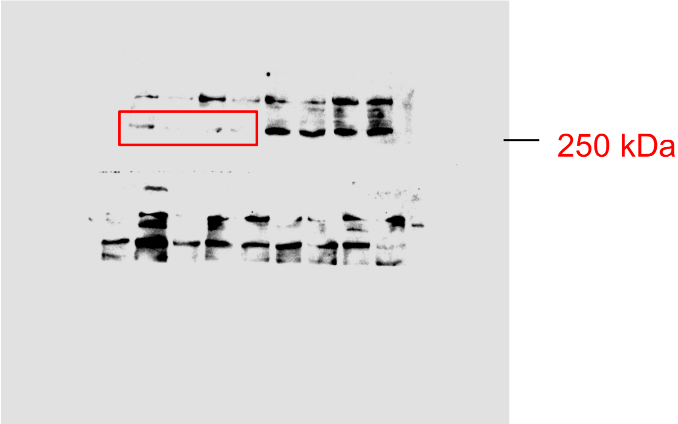

250 kDa

PARP

PARP*

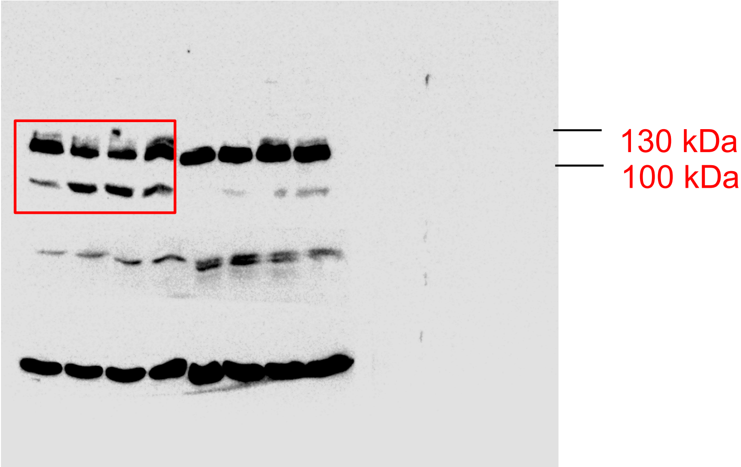

130 kDa
100 kDa

p53

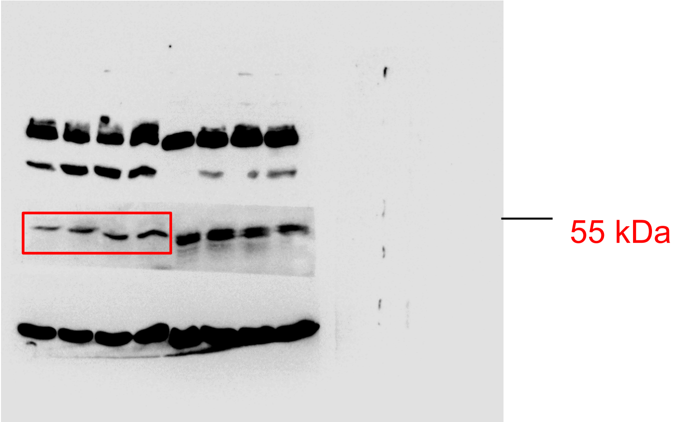

55 kDa

GAPDH

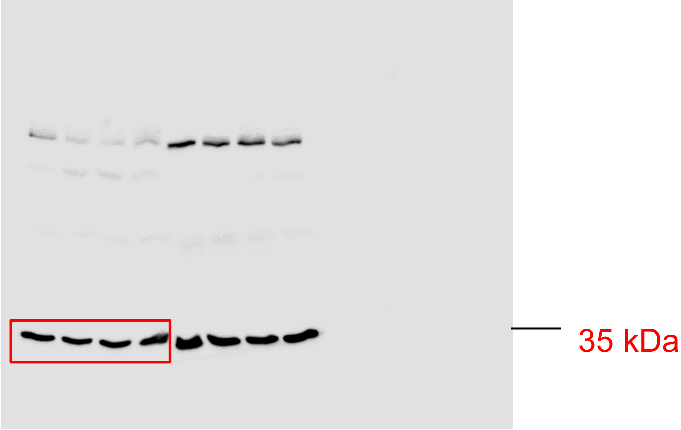

35 kDa

Source Data for Supplementary Fig. 5a

pFOXM1, CLB-BAR

pFOXM1, CLB-GE

FOXM1, CLB-BAR

FOXM1, CLB-GE

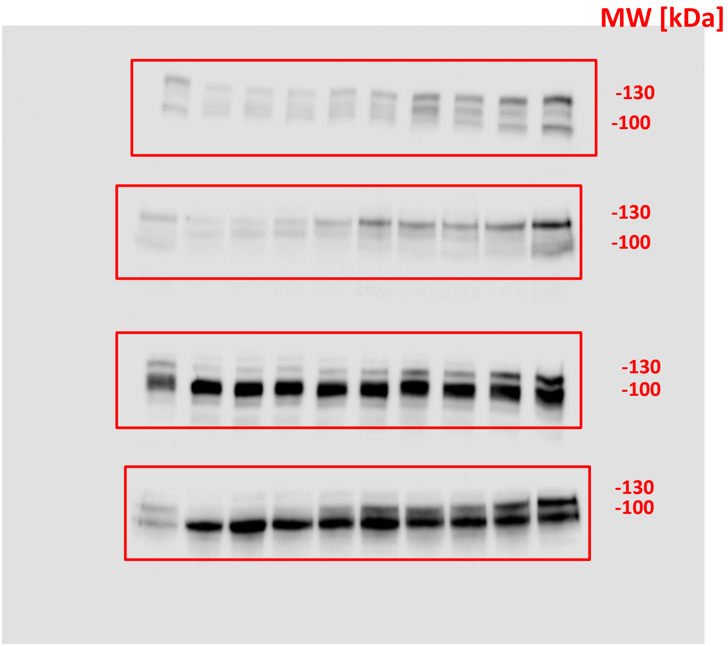

Actin, CLB-BAR

Actin, CLB-GE

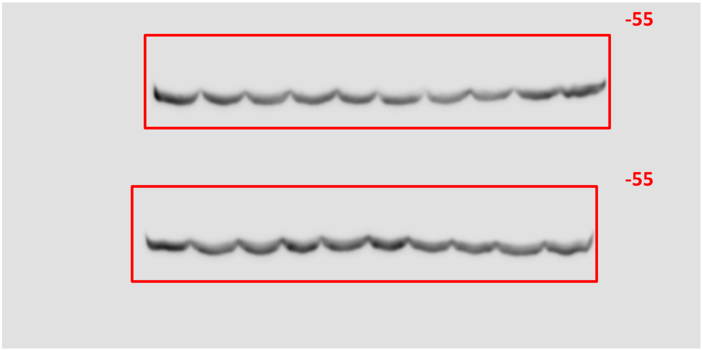

Source Data for Supplementary Fig. 5b

FOXM1

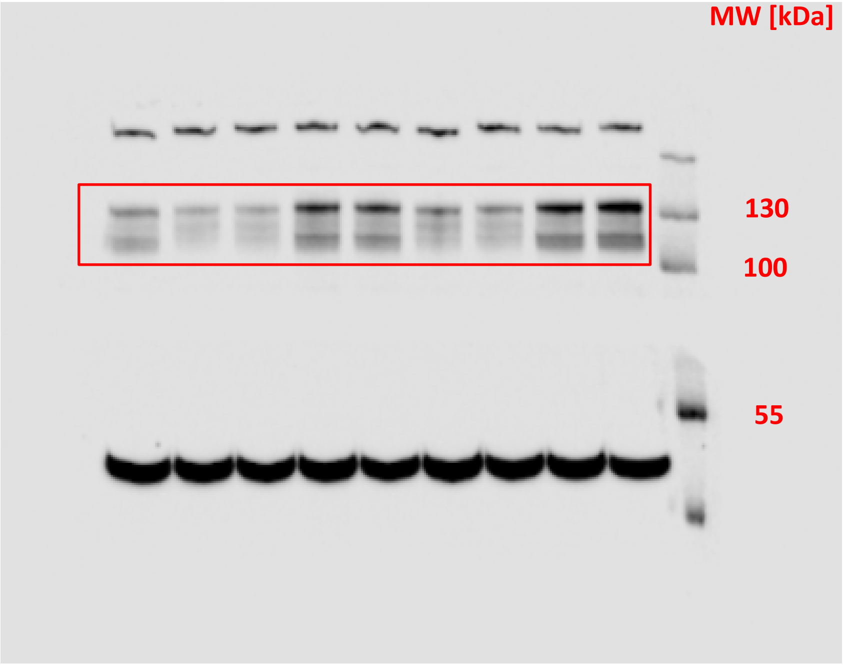

Actin

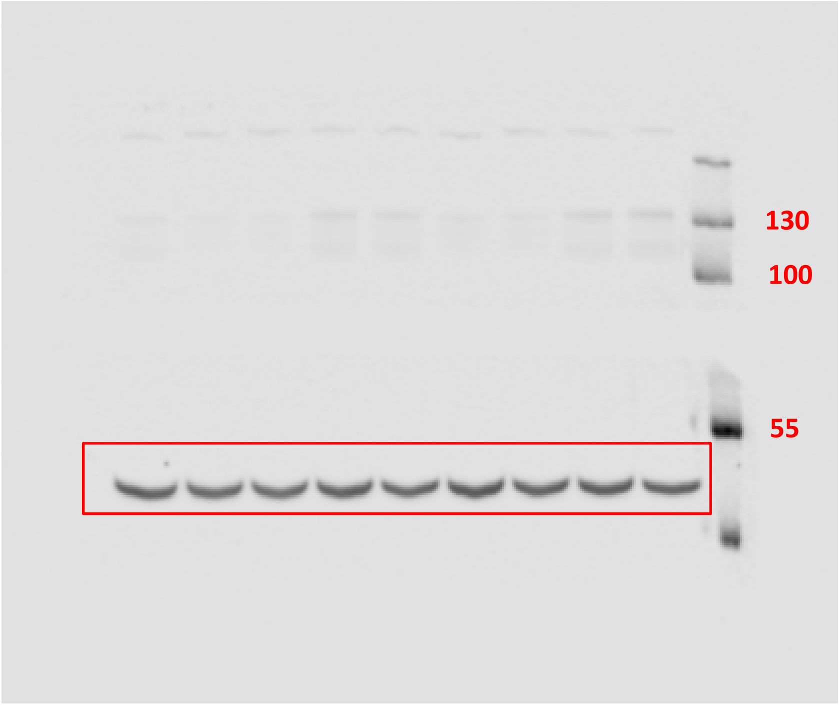

Supplement: Supplementary file 1 — Supplementary information file. [file 41467_2021_27057_MOESM1_ESM.pdf]
