# Supplementary material for: ATR inhibition enables complete tumour regression in ALK-driven NB mouse models
Source: Nat Commun. 2021 Nov 24;12:6813. doi: 10.1038/s41467-021-27057-2 (PMC8613282; doi:10.1038/s41467-021-27057-2)
Supplement: Supplementary file 2 — Description of Additional Supplementary files. [file 41467_2021_27057_MOESM2_ESM.docx]

**Description of Additional Supplementary Files**

**Title: Supplementary Dataset 1.**

Description: Overview of differential expression analysis for RNA-Seq, proteomics and phosphoproteomics performed in cell lines (first 3 tabs) and RNA-Seq in mice (last tab).

**Title: Supplementary Dataset 2.**

Description: Phosphoproteomics gene set enrichment analysis (GSEA). Canonical pathways GSEA was performed on 31 proteins containing hypophosphorylated sites and 417 proteins containing hyperphosphorylated sites as indicated by tab names. Pathways ordered according to enrichment p values with most significantly enriched pathways on top. GSEA was perfomed using Fisher’s exact test followed by false discovery rate (FDR) correction using the Benjamini-Hochberg method.

**Title: Supplementary Dataset 3.**

Description: Proteomics and phosphoproteomics quantification data. First 2 tabs contain the proteomics data for CLB-GE and CLB-BAR cells as indicated. Tab 3 contains the phosphoproteomics data for CLB-BAR cells. Protein identification FDR confidence indicated by first column: high confidence defined at 1% FDR and medium confidence at 5% FDR. FDR calculated using the Benjamini-Hochberg method.
