# Supplementary material for: ATR inhibition enables complete tumour regression in ALK-driven NB mouse models
Source: Nat Commun. 2021 Nov 24;12:6813. doi: 10.1038/s41467-021-27057-2 (PMC8613282; doi:10.1038/s41467-021-27057-2)
Supplement: Supplementary file 6 — Reporting summary. [file 41467_2021_27057_MOESM6_ESM.pdf]

nature portfolio

Corresponding author(s): *Ruth Palmer, Jimmy Van den Eynden, Bengt Hallberg*

Last updated by author(s): 2021-10-23

Reporting Summary

Nature Portfolio wishes to improve the reproducibility of the work that we publish. This form provides structure for consistency and transparency in reporting. For further information on Nature Portfolio policies, see our Editorial Policies and the Editorial Policy Checklist.

Statistics

For all statistical analyses, confirm that the following items are present in the figure legend, table legend, main text, or Methods section.

n/a	Confirmed	
☐	☒	The exact sample size (*n*) for each experimental group/condition, given as a discrete number and unit of measurement
☐	☒	A statement on whether measurements were taken from distinct samples or whether the same sample was measured repeatedly
☐	☒	The statistical test(s) used AND whether they are one- or two-sided<br>*Only common tests should be described solely by name; describe more complex techniques in the Methods section.*
☒	☐	A description of all covariates tested
☐	☒	A description of any assumptions or corrections, such as tests of normality and adjustment for multiple comparisons
☐	☒	A full description of the statistical parameters including central tendency (e.g. means) or other basic estimates (e.g. regression coefficient) AND variation (e.g. standard deviation) or associated estimates of uncertainty (e.g. confidence intervals)
☐	☒	For null hypothesis testing, the test statistic (e.g. *F*, *t*, *r*) with confidence intervals, effect sizes, degrees of freedom and *P* value noted<br>*Give P values as exact values whenever suitable.*
☒	☐	For Bayesian analysis, information on the choice of priors and Markov chain Monte Carlo settings
☒	☐	For hierarchical and complex designs, identification of the appropriate level for tests and full reporting of outcomes
☐	☒	Estimates of effect sizes (e.g. Cohen's *d*, Pearson's *r*), indicating how they were calculated

Our web collection on statistics for biologists contains articles on many of the points above.

Software and code

Policy information about availability of computer code

Data collection	All data were generated from cell line and mice experiments as described in the manuscript. The source code and processed data used for the bioinformatics analysis are available via Github https://github.com/CCGGlab/ATR and https://doi.org/10.5281/zenodo.5594083.
Data analysis	Data were analysed using R version 4.0 and CRAN/Bioconductor packages igraph (1.2.6), DESeq2 (1.30.0), DEP (1.12.0), protr (1.6.2) and fgsea (1.16.0). RNA-Seq data were analysed using hisat (2.1.0) and htseq (0.11.2) as described in methodology section.

For manuscripts utilizing custom algorithms or software that are central to the research but not yet described in published literature, software must be made available to editors and reviewers. We strongly encourage code deposition in a community repository (e.g. GitHub). See the Nature Portfolio guidelines for submitting code & software for further information.

Data

Policy information about availability of data

All manuscripts must include a data availability statement. This statement should provide the following information, where applicable:

- Accession codes, unique identifiers, or web links for publicly available datasets
- A description of any restrictions on data availability
- For clinical datasets or third party data, please ensure that the statement adheres to our policy

Data and code availability.
The mass spectrometry proteomics data are available in the ProteomeXchange Consortium via the PRIDE partner repository (dataset identifier PXD027187). RNA-seq data are available in ArrayExpress (https://www.ebi.ac.uk/arrayexpress/, accession numbers E-MTAB-10603 (https://www.ebi.ac.uk/arrayexpress/experiments/E-MTAB-10603/) & E-MTAB-10616 (https://www.ebi.ac.uk/arrayexpress/experiments/E-MTAB-10616/)). All other data required to evaluate the conclusions in the paper are provided in Supplementary information files. Source code used for RNA-Seq, proteomics and phosphoproteomics analyses is available at GitHub https://github.com/CCGGlab/ATR and https://doi.org/10.5281/zenodo.5594083.

Field-specific reporting

Please select the one below that is the best fit for your research. If you are not sure, read the appropriate sections before making your selection.

- [x] Life sciences
- [ ] Behavioural & social sciences
- [ ] Ecological, evolutionary & environmental sciences

For a reference copy of the document with all sections, see nature.com/documents/nr-reporting-summary-flat.pdf

RNA-Seq, Proteomics and Phospohr samples were performed with at least an n=3 (biological repeats). Detailed histological analyses of mice

Sample size

Life sciences study design

All studies must disclose on these points even when the disclosure is negative.

Sample size for each experiment is indicated in the figure legend. RNA-Seq, Proteomics and Phosphor samples were performed with at least an n=3 (biological repeats). Detailed histological analyses of mice was performed with n=4 or more. RNA-Seq from mouse tumours was performed with at least n=3 biological samples. Mouse tumour treatment was carried out with at least n=3. Other experiments, proliferation, viability, cell assays, were performed with n>3 or more, with the exception of the proteomics/phosphor samples that were performed as either biological duplicates/triplicates or quadriplets as specified. No statistical sample size calculation was performed to predetermine sample sizes. Sample size was chosen based on a balance between cost and replicate power for RNA-Seq, Proteomics and Phospohr samples. Sample sizes were based on previous experience with the experiments performed. All available mice were used to provide sufficient statistical power in the mouse experiments.

Data exclusions	Animals suffering from unrelated illnesses were removed in accordance with ethical regulations. These animals were excluded from analyses.
Replication	Cell experiments, including proliferation, viability, cell assays, were repeated >3 times. All experimental findings were successfully reproduced in multiple independent experiments. The number of independent experiments is indicated in the Figure legends.
Randomization	Animals were followed over time in a non-biased manner and treatment initiated on tumour detection. The order of tumour development determined the animals treatment with either vehicle or drug, irrespective of age and sex. Therefore, animals were randomly assigned to vehicle or drug treatment groups. For experiments involving cell lines samples were randomly allocated into treatment groups.
Blinding	Mouse treatment was performed on mice presenting with tumors following strict ethical procedures in a non-blinded manner. Tumor imaging was performed by ultrasound in a blinded manner. Quantification of IHC analysis of mouse tumors was performed in a blinded manner. For in vitro proliferation experiments, investigators were blinded for data acquisition and analysis. For routine cellular molecular biology experiments, investigators were not blinded as handling of cells, preparation of reagents and treatments required accurately recorded information.Proteomics/ phosphoproteomics was performed at the University of Gothenburg Core facility in a blinded manner and resulting data decoded by the respective investigators and bioinformatitions.

Reporting for specific materials, systems and methods

We require information from authors about some types of materials, experimental systems and methods used in many studies. Here, indicate whether each material, system or method listed is relevant to your study. If you are not sure if a list item applies to your research, read the appropriate section before selecting a response.

Materials & experimental systems

n/a	Involved in the study	
[ ]	[x]	Antibodies
[ ]	[x]	Eukaryotic cell lines
[x]	[ ]	Palaeontology and archaeology
[ ]	[x]	Animals and other organisms
[x]	[ ]	Human research participants
[x]	[ ]	Clinical data
[x]	[ ]	Dual use research of concern

Methods

n/a	Involved in the study	
[x]	[ ]	ChIP-seq
[x]	[ ]	Flow cytometry
[x]	[ ]	MRI-based neuroimaging

Antibodies

Antibodies used

Primary antibodies against ATR (#13934, 1:1000), pATR (s428, #2853, 1:1000), ATM (#2873, 1:1000), pATM (#13050, 1:1000), CHK1(#2345, 1:1000), pCHK1(S345,#2348, 1:1000), pAKT (S473,#4060, 1:4000), pERK1/2 (Y204/T202, #4377, 1;2000), pALK (Y1604, #3341, 1:1000), β-Actin (#4970, 1:10000), AKT (#9272), FOXM1 (#D3F2B, 1:1000), pFOXM1 (T600, #14655, WB 1:100, IHC 1:1000), Ki67 (#12202, 1:800), -tubulin (#2125), cleaved caspase-3 (#9661, 1:200), p21 (#2947, 1:50), survivin (#2808, 1:400), phosphor histone H3 (9701, 1:200), GAPDH (#5174, 1:20000), p53 (#2527, 1:1000) and PARP (#9542, 1:1000) were obtained from Cell Signaling Technology. Phospho-ATM/ATR Substrate Motif [(pS/pT) QG] MultiMab™ Rabbit mAb mix (#6966) was also from Cell Signaling Technology. Primary antibodies detecting SUN2 (ab124916, 1:5000), CD68 (#ab125212; 1:100), and TOP2A (#ab52934, 1:1000) were purchased from Abcam and from Atlas Antibodies (HPA001209). Pan-ERK1/2 antibody (#610123, 1:10000) was purchased from BD Transduction Laboratories (Franklin Lakes, NJ). Monoclonal antibody 135 (anti-ALK, 1:100) was produced in-house against the extracellular domain of ALK66. Horseradish peroxidase -conjugated secondary antibodies, goat anti-mouse immunoglobulin G (IgG) (# 32230), and goat anti-rabbit IgG (# 32260, 1:5000) were from Thermo Fisher Scientific.

Validation

Antibody validations and validation criteria are available at the following websites:
Anti-ATR (#13934), https://www.cellsignal.com/products/primary-antibodies/atr-e1s3s-rabbit-mab/13934;
Anti-pATR (s428, #2853 1:1000), https://www.cellsignal.com/products/primary-antibodies/phospho-atr-ser428-antibody/2853;
Anti-ATM (#2873), https://www.cellsignal.com/products/primary-antibodies/atm-d2e2-rabbit-mab/2873;
Anti-pATM (#13050), https://www.cellsignal.com/products/primary-antibodies/phospho-atm-ser1981-d25e5-rabbit-mab/13050;
Anti-CHK1(#2345), https://www.cellsignal.com/products/primary-antibodies/chk1-antibody/2345;
Anti-pCHK1(S345,#2348), https://www.cellsignal.com/products/primary-antibodies/phospho-chk1-ser345-133d3-rabbit-mab/2348;
Anti-pAKT (S473,#4060), https://www.cellsignal.com/products/primary-antibodies/phospho-akt-ser473-d9e-xp-rabbit-mab/4060;
Anti-pERK1/2 (Y204/T202, #4377), https://www.cellsignal.com/products/primary-antibodies/phospho-p44-42-mapk-erk1-2-thr202-tyr204-197g2-rabbit-mab/4377;
Anti-pALK (Y1604, #3341), https://www.cellsignal.com/products/primary-antibodies/phospho-alk-tyr1604-antibody/3341;
Anti-β-Actin (#4970), https://www.cellsignal.com/products/primary-antibodies/b-actin-13e5-rabbit-mab/4970;
Anti-AKT (#9272), https://www.cellsignal.com/products/primary-antibodies/akt-antibody/9272;
Anti-FOXM1 (#20459), https://www.cellsignal.com/products/primary-antibodies/foxm1-d3f2b-rabbit-mab/20459?site-search-type=Products&N=4294956287&Ntt=d3f2b&fromPage=plp;
Anti-pFOXM1 (T600, #14655), https://www.cellsignal.com/products/primary-antibodies/phospho-foxm1-thr600-d9m6g-rabbit-mab/14655;
Anti-Ki67 (#12202), https://www.cellsignal.com/products/primary-antibodies/ki-67-d3b5-rabbit-mab-mouse-preferred-ihc-formulated/12202;
Anti-tubulin (#2125), https://www.cellsignal.com/products/primary-antibodies/a-tubulin-11h10-rabbit-mab/2125;
Anti-cleaved caspase-3 (#9661), https://www.cellsignal.com/products/primary-antibodies/cleaved-caspase-3-asp175-antibody/9661;
Anti-p21 (#2947), https://www.cellsignal.com/products/primary-antibodies/p21-waf1-cip1-12d1-rabbit-mab/2947;
Anti-survivin (#2808), https://www.cellsignal.com/products/primary-antibodies/survivin-71g4b7-rabbit-mab/2808;
Anti-phosphor histone H3 (#9701), https://www.cellsignal.com/products/primary-antibodies/phospho-histone-h3-ser10-antibody/9701;

Anti-GAPDH (#5174), https://www.cellsignal.com/products/primary-antibodies/gapdh-d16h11-xp-rabbit-mab/5174;
Anti-p53 (#2527), https://www.cellsignal.com/products/primary-antibodies/p53-7f5-rabbit-mab/2527;
Anti-PARP (#9542), https://www.cellsignal.com/products/primary-antibodies/parp-antibody/9542;
Anti-SUN2 (#ab124916), https://www.abcam.com/sun2-antibody-epr6557-ab124916.html;
Anti-CD68 (#ab125212), https://www.abcam.com/cd68-antibody-ab125212.html;
Anti-TOP2A (#ab52934), https://www.abcam.com/topoisomerase-ii-alpha-antibody-ep1102y-ab52934.html;
Anti-Pan-ERK1/2 antibody (#610123), https://www.bdbiosciences.com/en-us/products/reagents/microscopy-imaging-reagents/immunofluorescence-reagents/purified-mouse-anti-erk-pan-erk.610123
Monoclonal antibody 135 (anti-ALK) has been validated in Guan et al, PMID: 28030793, 2017.

Eukaryotic cell lines

Policy information about cell lines

Cell line source(s)	NB cell lines CLB-BAR, CLB-GE, CLB-GAR, SK-N-AS, IMR32, NB-1, SKSY5Y, Kelly and SK-N-BE(2) were employed in this study (genetic details are provided in Fig S1). CLB-BAR, CLB-GE and CLB-GAR were obtained from The Centre Leon Berard, France under MTA. All other cell lines are from ATCC.
Authentication	Cell lines have been authenticated by STR profiling in the last 24 months.
Mycoplasma contamination	Cell lines are regularly tested for mycoplasma contamination. All cell lines used in this study tested negative for mycoplasma contamination.
Commonly misidentified lines (See ICLAC register)	No commonly misidentified cell lines were used in the study.

Animals and other organisms

Policy information about studies involving animals; ARRIVE guidelines recommended for reporting animal research

Laboratory animals	Alk-F1178S mice were custom generated by PolyGene Transgenetics (Polygene AG, Switzerland) (Borenas, EMBO J, 2021). Rosa26_Alkal2 mice were custom generated by Ozgene Pty Ltd (Bentley DC, Australia) (Borenas, EMBO J, 2021). The above mice were backcrossed to a 129X1/SvJ background. Th-MYCN mice (Weiss et al., 1997) were on genetic background 129X1/SvJ. A mixture of gender and ages of mice were used, as the main criteria was tumour presentation. Mice were kept in a controlled enviroment within the University of Gothenburg animal facility with free access to both water and food and in accordance with ethical regulations.
Wild animals	No wild animals samples were used in the study.
Field-collected samples	No field collected samples were used in the study.
Ethics oversight	All experimental procedures and protocols were performed in accordance with the Regional Animal Ethics Committee approval, Jordbruksverket (1890-2018).

Note that full information on the approval of the study protocol must also be provided in the manuscript.
